# Locus-specific endogenous retroviral transcriptional modulation in viral hepatitis–driven hepatocellular carcinoma

**DOI:** 10.1016/j.tvr.2026.200352

**Published:** 2026-10-29

**Authors:** Nicole Grandi, Elena Soddu, Saili Chabukswar, Sante Scognamiglio, Yun Yen, Ching-Hsuan Liu, Liang-Tzung Lin, Enzo Tramontano

**Affiliations:** a Laboratory of Molecular Virology, Department of Life and Environmental Sciences, University of Cagliari, Italy; b Graduate Institute of Cancer Biology and Drug Discovery, College of Medical Science and Technology, Taipei Medical University, Taipei, Taiwan; c Graduate Institute of Pharmacognosy, College of Pharmacy, Taipei Medical University, Taipei, 110301, Taiwan; d Department of Microbiology and Immunology, School of Medicine, College of Medicine, Taipei Medical University, Taipei, Taiwan; e Graduate Institute of Medical Sciences, College of Medicine, Taipei Medical University, Taipei, Taiwan

**Keywords:** Hepatocellular carcinoma, HERV, Endogenous retroviruses, Transcriptomic, Hepatitis

## Abstract

Human endogenous retroviruses (HERVs) are remnants of ancient infections integrated into the primate germ line. HERV transcriptional reactivation has been observed in several diseases, including cancer, where it may reflect epigenetic deregulation, cellular stress, inflammation, and infections. Hepatocellular Carcinoma (HCC) is one of the most diffuse and deadly cancers worldwide, with chronic hepatitis infections as major etiological factors. In this context, the activation of specific HERV loci in HCC and their relationship with the broader tumor-associated transcriptional landscape remain poorly defined. Here, we characterized the expression of ∼3300 HERVs in a public RNA-seq dataset comprising HCC samples, predominantly of viral etiology, and non-HCC liver controls. We identified 185 differentially expressed HERVs, predominantly upregulated in HCC (n = 145): comparisons with GTEx healthy liver transcriptomes, inflammatory gene profiling, and covariate-adjusted analyses supported the consistency of this HCC-associated transcriptional signature. Aggregate expression scores of upregulated and downregulated HERVs showed significant associations with histological grade, with opposite trends across G1–G3 tumors. 16 HERV loci were consistently upregulated in all tumor tissues, and their aggregate expression increased significantly with histological grade, supporting their prioritization as a candidate HCC-associated signature. HERV-correlated pathway analyses revealed grade-associated patterns involving metabolic, inflammatory, interferon-related, and proliferative transcriptional programs. De novo transcript reconstruction identified candidate HERV-derived transcripts requiring experimental validation. Overall, the study provides a comprehensive characterization of HERV modulation in a predominantly viral-associated HCC cohort and identifies conserved HERV signatures embedded within the broader transcriptomic remodeling, for future independent and functional studies of their potential relevance to liver cancer biology.

## 1 Introduction

Hepatocellular carcinoma (HCC) is the most common primary liver cancer and, in 2022, it was the third leading cause of cancer deaths worldwide with a relative 5-year survival rate of approximately 15-20% [1], [2]. The most important risk factors for HCC remain the chronic hepatitis caused by hepatitis B virus (HBV) and hepatitis C virus (HCV), alcohol-related liver disease, and metabolic dysfunction-associated steatotic liver disease (MASLD). In the future, a possible change in this scenario could be due to the availability of DAA (Direct-Acting Antivirals) and vaccines against viral hepatitis, which, together with the increase in bad lifestyle habits, could make MASLD the most relevant factor [1], [3], [4].

Chronic HBV infection is associated with a 5-100 times higher risk of developing HCC [5], [6]. HBV mechanisms to induce HCC involve integrated viral genome instability, Wnt/β-catenin and PI3K/Akt pathways activation, methylation and histone changes, immune suppression, viral proteins-driven oxidative stress, and MAPK14 activation [6]. Chronic HCV infection enhances the risk to develop HCC of 15–30 times. Viral mechanisms associated with liver damage involve rapid viral mutation, IFN pathways suppression, immune cell exhaustion, and hepatic stellate cells activation and subsequent onset of fibrosis and cirrhosis [7]. Concern about HCC's is increased by difficulties in efficient early-stage diagnosis, lack of effective therapies, and low rates of overall survival without relapse [8]. These issues have stimulated efforts to uncover suitable biomarkers and novel drug targets, also exploring non-traditional approaches. In this context, Human Endogenous Retroviruses (HERVs) have acquired attention, as their possible involvement is already under investigation for many liquid and solid cancers [8], [9], [10].

HERVs are remnants of ancient infections by extinct retroviruses that occurred mostly 60 to 20 million years ago, with some groups - such as the HERV-K(HML2) clade - showing recent activity in humans until 0.2 million years ago [10]. Such infections led to the massive integration of proviral DNA into the genome of primate germ line cells along evolution, allowing their stable acquisition and vertical transmission through the offspring, and constituting today over 8% of the human genome [9], [10]. HERVs are expressed in various human tissues under physiological conditions, and their transcriptional modulation has been observed in pathological settings. Such modulation could be induced by the deregulation of transcriptional silencing mechanisms, as frequently observed in tumor tissues, as well as due to various external stimuli, including chemical substances, physical agents, and exogenous viral infections [11].

Evidence of HERVs’ modulation has been reported in leukemia, lung cancer, breast cancer, prostate cancer, and colorectal cancer [8], [10], among others. Nevertheless, the precise role of HERV expression in carcinogenesis and the eventual molecular mechanisms of contribution to the disease are still debated, also due to difficulties in distinguishing whether the observed HERV activation is a pathological driving force or a simple bystander consequence of tumorigenesis [8], [9], [10]. The residual production of HERV envelope proteins (Env) can directly support tumorigenesis, stimulating cellular fusion and genetic instability. In this regard, Syncytin-1 (Syc-1) is the most relevant example due to its known fusogenic activity, involved in placenta physiology and investigated for its role in tumorigenesis [10]. A direct oncogenic potential has also been observed for HERV-K(HML2) Env splicing variants Np9 and Rec, both expressed in different tumor tissues [10], [11]. In addition, HERVs could support oncogenesis by activating specific tumor-promoting cellular pathways through their Long Terminal Repeats (LTRs), which might serve as alternative promoters or enhancers for cellular genes involved in tumorigenesis, providing alternative binding sites for transcription factors or other regulatory proteins [10], [11]. Finally, HERV transcription can produce non-coding RNAs affecting the regulation of co-localized genes, possibly leading to the uncontrolled activation of oncogenes or the silencing of oncosuppressors [9], [10]. A similar role in tumor development might also be provided by HERV RNA included in retroviral‐like microvesicles, as found in patients with lymphomas, breast cancer, melanoma, and teratomas [11]. Notably, all these mechanisms can be supported by the altered epigenetic environment of tumor tissues, in which DNA hypomethylation and chromatin remodeling are often associated with HERV transcriptional de-repression along tumorigenesis [11], [12].

In recent years, the potential contribution of HERV expression in HCC has gained growing attention [8]. The available evidence suggests a correlation between HERV modulation and HCC patients' prognosis, tumor progression, and overall survival [13], [14], [15]. For instance, HERV-K(HML2) upregulation might be correlated to cirrhosis, histological grade, and tumor stage [13], [14]. Similarly, the HERV-H LTR-associating 2 (HHLA2) is an immune checkpoint protein that may be correlated to HCC recurrence, metastasis, and poor prognosis, being involved either in tumor immune evasion or cellular inflammatory responses associated with tumor development, such as TNF and Toll-like receptor signaling pathways [16]. In addition, the modulation of HERV LTR families - such as LTR1, LTR7, LTR12C, and THE1B - has also been observed in HCC, possibly contributing to carcinogenesis through their promoter or enhancer activity, as described above [17]. Of note, HCC tissues characterized by high levels of LTR expression showed a higher risk of relapse, aggressive phenotype, and poor prognosis [8] Interestingly, the 5′LTR of the HERV-K(HML6) locus 16p11.2 overlaps with the first exon of a transcript of the ZNF689 gene, observed as overexpressed in HCC, that encodes for a zinc finger protein involved in suppressing apoptosis in the tumor [18], [19], further suggesting the possible interplay between HERV regulation and oncogene expression. Concerning HERV proteins, Syc-1 resulted upregulated in HCC in vitro models [8], [20] being correlated with the induction of the MEK/ERK pathway which is in turn often activated in HCC and associated with its development [8]. Finally, some studies assessed the interplay between HERV deregulation and the presence of HBV and HCV infections, the first risk factors of HCC [14], [21], [22]. HBV encoded X protein (HBx), an important regulator of viral-induced HCC, has been suggested to increase the promoter activity of Syc-1 LTR through the NF-κB pathway [14]. Interestingly, Syc-1 upregulation has been observed in HepG2 cells - a HCC model transfected with HBx, confirming the activation of the related HERV-W locus by HBx [8], [14], [22]. In the context of HCV infection, higher transcription levels of HERV-H and HERV-K *pol* genes have been reported in infected individuals, before and after DAAs therapy, as compared to controls [21]. Although HCC presence was not considered in the mentioned study, it raises attention on how such HERV genes could be involved in higher cancer risk in DAAs-driven infection-recovered patients [21].

While the above-described studies reported evidence for HERV involvement in HCC, such results are significantly limited by the fact that they were conducted either on whole HERV groups, without identifying the specific members actually modulated, or on single HERV sequences (Syc-1), without characterizing exhaustively all the specific HERV loci that are upregulated or repressed in the presence of the infection and in the consequent tumoral transformation. Moreover, such studies analyzed HERV modulation considering either tumor presence or viral infection.

To investigate the transcriptional modulation of HERVs in virus-associated HCC and their relationship with the broader tumor transcriptomic landscape, we analyzed the expression of ∼3300 HERV loci and ∼66,000 cellular genes in 61 HCC liver tissue samples, predominantly composed of viral-associated HCC, and 15 non-tumoral (NT) liver samples from a publicly available RNA-seq dataset [23]. The study first characterized global HERV and cellular gene modulation associated with HCC, while evaluating the potential influence of cohort composition, inflammatory signatures, and clinical covariates on the observed transcriptional patterns. We then investigated HERV modulation according to HCC histological grade and identified a set of 16 HERV loci consistently upregulated across all samples. Finally, these conserved HERV loci were further characterized through pathway enrichment analyses, transcript reconstruction, genomic contextualization, and coding-potential assessment to identify specific elements with transcriptional features warranting more detailed investigation.

## 2 Materials and methods

### 2.1 RNA-seq dataset

The RNA-seq dataset analyzed includes transcriptomic profiles from liver tumor tissues of a cohort of HCC patients (61), mostly with viral etiology, and non-tumoral (NT) liver samples (15) from patients affected by cholangiocarcinoma or polycystic liver disease (non-infected). The dataset has been obtained from the study of Sang-Ho Yoon et al. [23] through the Gene Expression Omnibus (GEO) Databank with the accession number GSE148355 [24]. The dataset raw data have been downloaded as fastq files from the European Nucleotide Archive (ENA) [25] and included an average of 56 million paired-end reads of 100 + 100 nucleotides.

### 2.2 Data processing

The quality check of each fastq file has been performed with the FastQC software, followed by a trimming procedure with the Trim Galore tool. Processed reads have been mapped to the reference human genome assembly GRCh38/hg38 with the STAR aligner, version 2.5.2 [26]. The quantification of the reads mapping to specific HERV loci (∼3300 HERVs, in-house database) and cellular genes (∼66,000, Gencode dataset version 38) was performed with the HTSeq-count tool, relying on HERV and genes' univocal genomic coordinates [27]. The obtained raw counts have been normalized by regularized-logarithm transformation, considering each HERV and gene length and the samples’ sequencing depth, and used for unsupervised clustering and differential expression analysis. The same raw counts have been used to calculate the relative abundance of reads as transcripts per million Kb (TPM).

### 2.3 Unsupervised clustering and differential expression analysis

Unsupervised Clustering analysis has been performed with the DESeq2 package (version 1.46.1) [28] with RStudio software, version 2024.12.1 + 563 [29] and the related plots generated with gplot (version 3.1.3.), ggplot2 (version 3.5.1.), RcolorBrewer (version 1.1-3), and pheatmap (version 1.0.12) packages.

The DESeq2 package has been used for the DE analyses [28], setting as a threshold an absolute log2foldChange (log2FC) ≥ 1 and an adjusted p-value (p-adj) ≤ 0.01. Each DE HERV and gene has been classified as upregulated (DEL+) with log2FC ≥ 1 and downregulated with log2FC ≤ −1. Results have been visualized as Volcano plots and Venn diagrams generated with the packages ggplot2 (version 3.5.1.) and VennDiagram (version 1.7.3), respectively.

Gene enrichment analysis has been performed through the integrative web-based and mobile software application Enrichr [30] applying the available library GO Biological Process 2025 [31], setting as threshold for enrichment a p-adj ≤ 0.01. The related plots have been generated through the R packages ggplot (version 3.5.1.) and ComplexHeatmap (2.22.0).

### 2.4 Histological grade-associated HERV expression analysis

To assess whether the overall HCC-associated HERV transcriptional signatures varied across histological grades, for each sample, HERV expression values were standardized as Z-scores, and the mean Z-score across all loci within each set was calculated to obtain an aggregate score. Differences among G1, G2, and G3 tumors were evaluated using the Kruskal–Wallis test, while monotonic trends across the ordinal NT–G1–G2–G3 groups were assessed using Spearman rank correlation.

### 2.5 Genomic context of HERV integration

HERV loci colocalization with cellular genes has been assessed on UCSC Genome Browser after the intersection of their genomic coordinates with the ones of Gencode (version 47), with the Data Integrator function [32]. HERV orientation and exonic or intronic localization with respect to the colocalized gene have been annotated.

### 2.6 Transcriptome reconstruction

Raw reads have been used for a *de novo* transcriptome reconstruction with the Trinity Toolkit [33]. HCC and NT samples have been merged and used to generate a transcriptome representative of tumoral and non-tumoral conditions, respectively. The transcriptomes have been mapped back to the human genome using the GMAP mRNA aligner [34] and visualized through the Integrative Genomics Viewer software (IGV) [24], [35]. Transcripts’ level of expression has been quantified through the Salmon tool [36]. The reconstructed transcripts were translated and aligned with their respective DFAM reference protein sequences using the MAFFT plugin in Geneious Prime 2026.01.1 software [37].

### 2.7 Pathway enrichment

To identify pathways associated with HERV loci, Spearman's rank correlations were calculated between each HERV locus and the entire protein-coding gene set of Gencode 47 using TPM values across all samples, as reported in Viot et al. [38]. Based on the correlation coefficients, genes were ranked from the strongest positive to the strongest negative association for each HERV. All the human HALLMARK signatures were retrieved with the Msigdb R package, and gene set enrichment analyses were performed using the FGSEA R package. FGSEA was conducted using 10,000 permutations with Spearman correlation coefficients, and the pathways with adjusted p-value of < 0.05 were considered significantly enriched.

## 3 Results

### 3.1 RNA-seq dataset

The analyzed dataset was extracted from the study of Sang-Ho Yoon et al. [23] (GEO accession number GSE148355) and included 61 liver tissue samples from HCC patients. The HCC cohort consisted predominantly of male patients (52, male-to-female ratio of ≈6:1) and showed a mean and median age of 55 and 59 years, respectively (range: 36-78 years) (Table 1). The dataset also provided information about tumor histological grading, i.e., G1, G2, and G3 [39], and 6 of the HCC patients also had comorbidity with ICC (Intrahepatic Cholangiocarcinoma). Tumors were mostly associated with viral hepatitis etiology (36 HBV- and 6 HCV), except for one alcohol-mediated HCC and 2 MASLD-associated cases (annotated as NASH in GSE148355 [24]), while the remaining 9 HCC samples had unknown etiology (Supplementary Table S1). The HCC cohort was compared to a non-tumoral (NT) cohort extracted from the same dataset, which comprised 7 males and 5 females (male-to-female ratio of ≈1.4:1) plus 3 individuals with unspecified gender, showing a mean and median age of 55 and 56 years, respectively (range: 17-76 years) (Table 1). Importantly, although these NT controls did not present HCC or viral hepatitis, they were not derived from healthy liver tissue, including hepatic biopsies from individuals affected by cholangiocarcinoma and polycystic liver disease. This aspect was taken into consideration during downstream differential expression analyses and interpretation.

**Table 1:** **Description of GSE148355 dataset.** The main characteristics of the individuals providing tumoral and non-tumoral samples are reported, including age, gender, G1-G3 tumor grade, eventual presence of comorbidity with ICC, and tumor etiology. NR = not reported, NA = not applicable.

	Samples	Age	Gender	Tumor grade	Comorbidity w/ICC	HCC etiology
**HCC**	61	55 (mean) 59 (median)	9 F 52 M	10 G1 35 G2 10 G3 6 NA	6 55 NA	36 HBV 6 HCV 2 MASLD 1 alcohol 9 NR
**NT**	15	55 (mean) 56 (median)	5 F 7 M 3 NR	NA	NA	NA

### 3.2 Unsupervised Clustering Analysis

To obtain a first overview of sample distribution according to HCC condition, unsupervised clustering analyses were performed using normalized raw counts of reads mapping to HERV and gene genomic coordinates.

#### 3.2.1 Sample to sample distance plot

The sample-to-sample distance plot based on HERV expression largely clustered samples according to the presence or absence of HCC (Fig. 1a, left), except for one NT sample (SRR11517958) that grouped with HCC samples, and two HCC samples (SRR11518078, SRR11518079) that are in turn included among the NT ones. The sample-to-sample plot of gene expression confirms the clustering of the samples according to HCC presence, without any outlier (Fig. 1a, right). To investigate the atypical clustering observed in the HERV-based analysis for the above three samples, we examined the immune-related features reported in the original study. The NT sample clustering with HCC tissues (SRR11517958) showed MUM1+ plasma cell infiltration levels comparable to those observed in HCC samples. Similarly, the two HCC samples clustering with NT tissues (SRR11518078, SRR11518079) displayed FoxP3 regulatory T cells and MUM1+ plasma cells counts comparable to those of the NT samples. Overall, these observations suggest that the extent of immune infiltrates may contribute to shaping HERV transcriptional profiles in liver tissues. Clustering analysis also showed that tumor etiology does not substantially influence sample clustering according to HERV expression (Supplementary Fig. S1a), strengthening the hypothesis that tumor presence is the main factor driving sample segregation. Nevertheless, the unequal distribution of etiological categories and the lack of information for 9 HCC samples may represent a source of variability influencing clustering patterns.

**Fig. 1:**
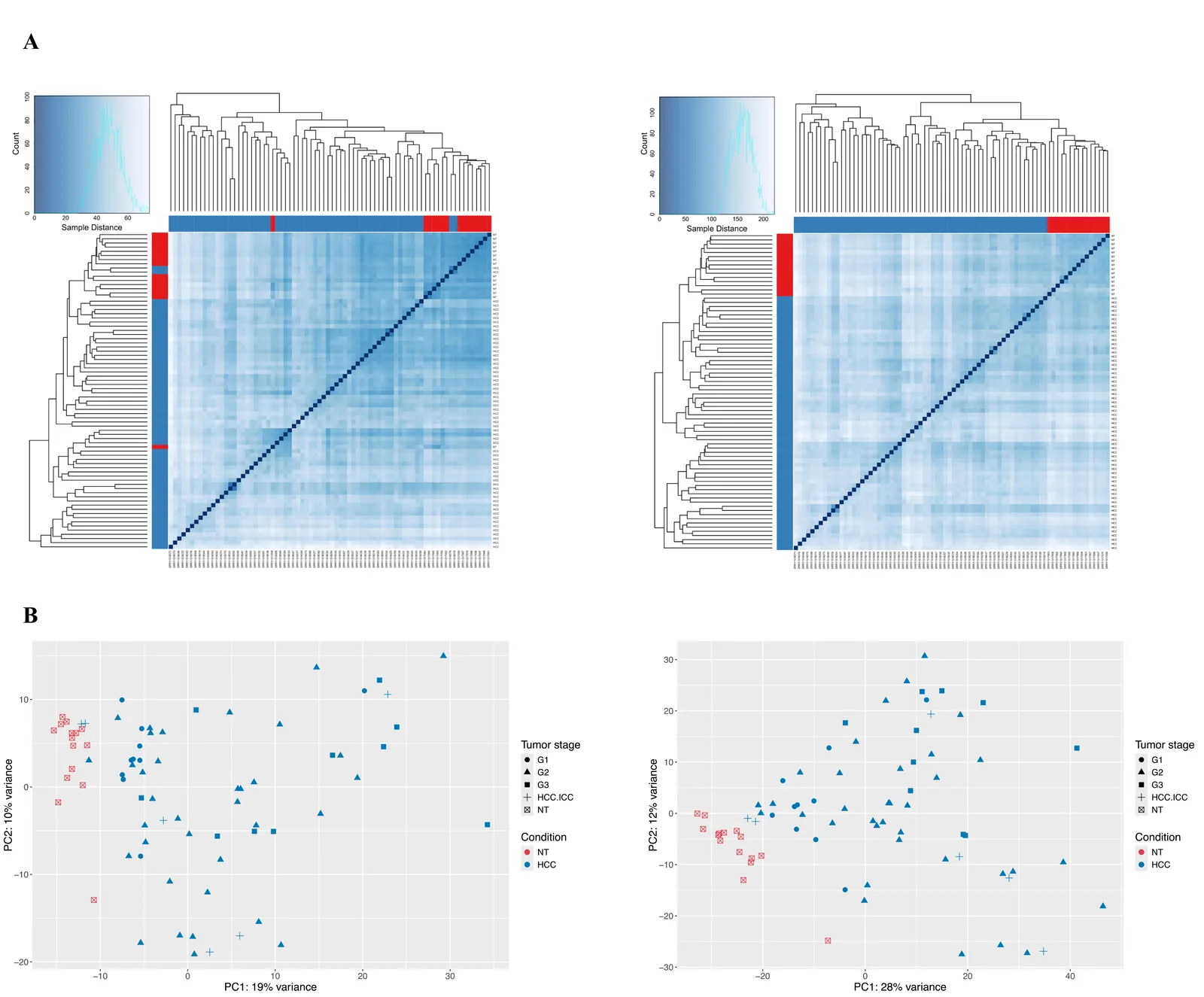
**Unsupervised Clustering Analysis.** Panel a) Sample-to-sample distance heatmaps have been generated according to pairwise distance in HERV (left) and gene expression (right) among the samples. The latter are classified as HCC (blue bars) and NT (red bars). Panel b) Principal Component Analysis (PCA) of HERV and gene expression variance. The first and second principal components (PC1 and PC2) that explain HERV (right) and gene (left) transcriptional variance are shown on the x-axis and y-axis, respectively. The samples are represented with different colors, accounting for tumor presence/absence, and shapes, accounting for G1-G3 grades and comorbidity with ICC.

#### 3.2.2 Principal components analysis (PCA)

PCA was performed to further evaluate sample distribution according to HERV and gene expression and to identify the principal components (PC1 and PC2) accounting for their variance (Fig. 1b). In the HERV-based PCA, PC1 explains 19% of HERVs’ transcriptional variance and does not completely divide samples according to HCC presence. Nevertheless, a tendency to such clustering is observed, with NT samples predominantly distributed on the left side of the plot and HCC ones localized mainly on the opposite side (Fig. 1b, left). Consistent with the sample-to-sample distance plot, the tumor etiology did not drive a specific grouping of the samples (Fig. S1b). However, the alcohol-induced HCC and one of the 2 MASLD-associated HCC samples clustered separately from the other HCC samples, suggesting that, as seen for immune-related profiles, metabolic alterations may also contribute to HERV transcriptional variability. Similarly, the PCA based on gene expression showed that PC1 (28% of variance) does not fully divide the samples according to HCC status but still revealed a tendency toward segregation between HCC and NT tissues (Fig. 1b, right). In both PCA plots, PC2 explained 10% to 12% of HERV and gene expression variance, respectively, and localized most (HERV) or all (genes) NT samples on the same side of the plot. Neither tumor histological grade nor ICC comorbidity showed a clear impact on the global HERV or gene expression variance (Fig. 1b).

#### 3.2.3 Expression heatmaps

Heatmaps based on the 500 HERVs and genes showing the highest expression variance were generated and further confirmed the segregation of the samples according to the presence (blue) or absence (red) of HCC (Fig. 2a). Consistent with the sample-to-sample distance analysis (Fig. 1a), the NT sample SRR11517958 clustered with the HCC group in both the HERV- and gene-based heatmaps, whereas the HCC samples SRR11518078 and SRR11518079 are grouped with the NT cohort (Fig. 2a). The heatmaps additionally revealed a subgroup of 21 HCC samples that clustered closer to the NT ones (Fig. 2a). This subgroup included 2 HCV- and 15 HBV-related tumors and 4 tumors with unknown etiology and comprised 8 of the 10 tumors assigned to the G1 histological grade, suggesting that well differentiated HCCs may retain expression profiles more similar to NT liver tissues.

**Fig. 2:**
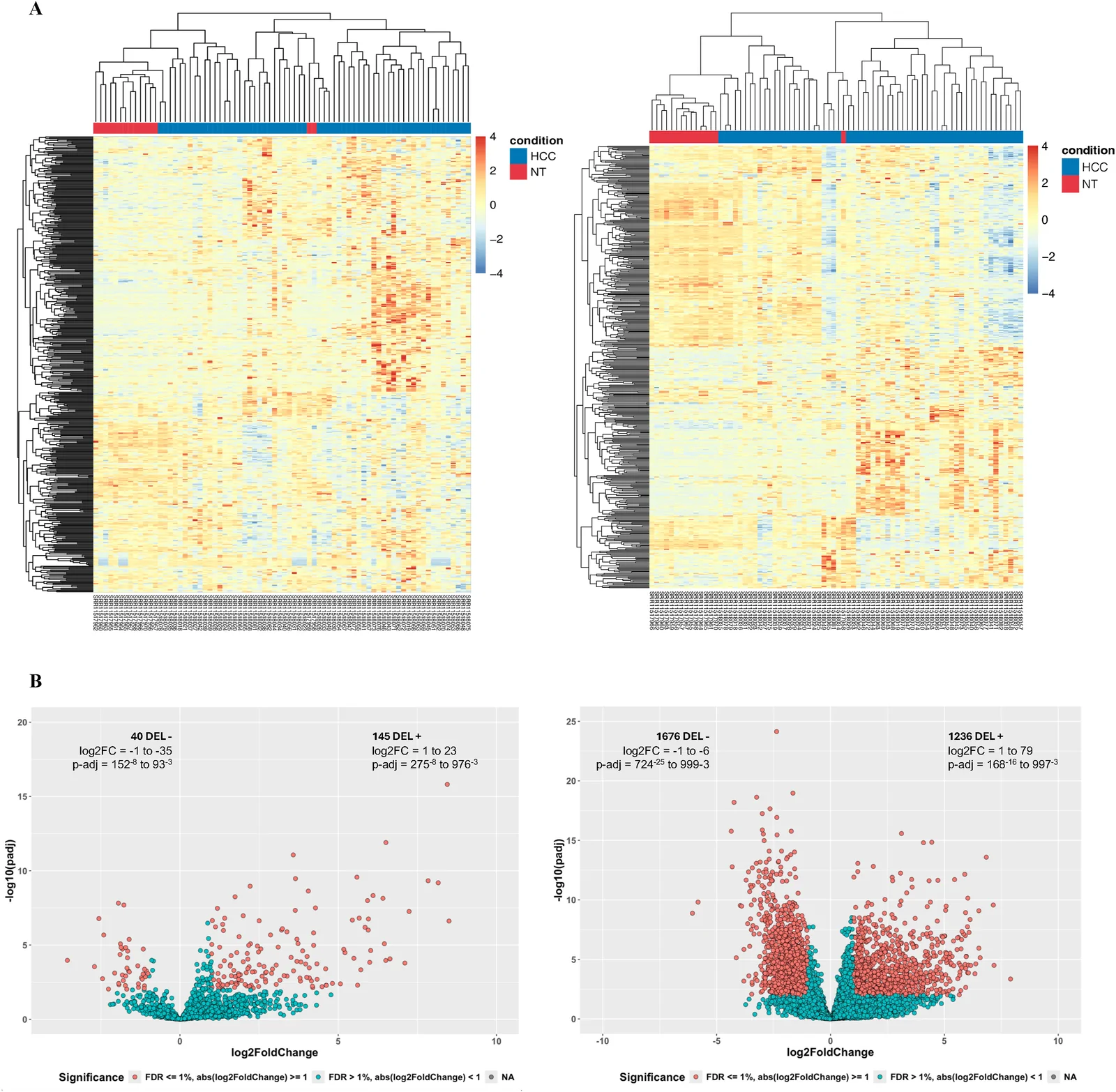
**Heatmaps of variance of expression.** Panel a) Heatmap of the 500 HERVs (left) and cellular genes (right) sorted by the highest variance of expression. The column dendrogram represents the patients' clustering, while the row dendrogram shows the clustering of HERVs and genes according to their expression in the different samples. Panel b) Volcano plots depicting HERV (left) and gene (right) differential expression results. Red dots represent elements modulated in HCC samples according to a p-adj ≤ 0.01 and a Log2Fold change ≤ −1 or ≥1, indicating downregulation or upregulation as compared to NT samples, respectively.

### 3.3 Differential expression analysis of HERVs and cellular genes

The DE analysis was first performed on the overall dataset to evaluate HERV and gene modulation in the HCC samples regardless of the tumor etiology, grade, or ICC comorbidity (Fig. 2b). 185 DE HERVs were identified, among which 145 were upregulated (DEL+) and 40 downregulated (DEL-). The gene analysis identified a total of 2912 DE elements in the tumoral samples, including 1236 DEL+ and 1676 DEL-genes. To further characterize the transcriptional landscape associated with HCC, subsequent analyses first focused on the validation and functional characterization of differentially expressed cellular genes, followed by the investigation of HERV modulation according to tumor etiology and histological grade.

#### 3.3.1 Validation of DE gene signature using healthy liver transcriptomes

Given the heterogeneous nature of the NT controls included in the analyzed cohort, we sought to evaluate whether the transcriptional modulation identified in HCC samples was also observed relative to healthy liver tissues. To this end, TPM values from 260 healthy liver samples were retrieved from the GTEx portal and used as an external reference dataset. Since the integration of independently generated RNA-seq datasets into a common differential expression pipeline may introduce substantial batch effects and technical biases, GTEx samples were not included in the DE analysis itself but were instead used as an external reference to compare the expression levels (as Transcripts per Million Kilobases, TPM) of DE genes across HCC samples, dataset-NT controls, and healthy liver tissues (GTEx-HC). Among the 2912 identified DE genes, a subset (n = 1277) displayed more evident transcriptional differences between the analyzed dataset and GTEx-HC samples. These genes formed two subsets according to their expression across samples: one displayed higher expression in HCC samples relative to both dataset-NT and GTEx-HC samples, which exhibited overall comparable expression levels (cluster 1), whereas the second showed increased expression in NT samples compared with both HCC and GTEx-HC tissues (cluster 2) (Supplementary Fig. S2a). To further characterize the genes enriched in dataset-NT samples (cluster 2), we performed functional enrichment analyses (Supplementary Fig. S2b). Notably, none of the identified biological processes remained significant after multiple testing correction, and the enriched categories were characterized by limited gene overlap, suggesting relatively modest transcriptional differences between dataset-NT and healthy liver tissues. The pathways showing nominal enrichment were mainly associated with nuclear protein import, cell cycle-related processes, and immune regulation. Finally, to evaluate whether the overall direction of differential expression observed in our study was maintained when compared with healthy liver tissues, we focused on the expression levels of the top 20 upregulated and top 20 downregulated DE genes identified in HCC (Supplementary Fig. S2c). Although some differences in absolute TPM values were observed between dataset-NT and GTEx-HC samples – particularly for downregulated genes – the top upregulated genes in HCC displayed overall comparable expression between the two control groups. Overall, these analyses supported the consistency of the transcriptional modulation observed in HCC relative to both dataset-NT and GTEx-HC controls.

#### 3.3.2 Assessment of inflammatory transcriptional signatures

Since differences in cellular composition and immune infiltration may also influence bulk transcriptomic profiles and HERV expression patterns, we next evaluated the distribution of immune- and inflammation-related transcriptional signatures across the analyzed cohorts. The potential impact of cellular composition and immune infiltration on transcriptomic profiles had already been extensively evaluated in the original study generating the analyzed dataset [23], which specifically focused on the immune landscape of premalignant and early HCC lesions through immunohistochemistry analyses and deconvolution-based approaches. Results identified increased immune infiltration in premalignant lesions adjacent to HCC tissues, followed by a reduction in established HCC samples, whose immune infiltration levels were reported to be overall comparable to those observed in the non-tumoral (NT) control group. Even if the present study reanalyzed only the HCC and NT subsets of the original cohort, i.e., the ones reported to have similar immune infiltrates, we sought to verify whether comparable inflammatory transcriptional profiles were maintained within the samples included in our analyses. To this end, we evaluated the expression levels of approximately 1100 immune- and inflammation-related genes retrieved from immuneDB across dataset-HCC, dataset-NT, and GTEx-HC (Supplementary Fig. S3). Heatmap analysis showed broadly comparable expression patterns between dataset-HCC and dataset-NT samples, supporting similar levels of immune-related transcriptional activity across the two groups. Some differences were observed relative to GTEx-HC tissues, likely reflecting both biological variability and inter-dataset technical effects. A subset of GTEx-HC samples clustered closely with dataset-derived samples, further supporting partial overlap in inflammatory transcriptional signatures between HCC and NT tissues. Overall, while these analyses do not exclude a contribution of cellular composition to the observed transcriptional variability, they suggest that broad differences in immune infiltration are unlikely to account for the HERV modulation patterns identified in this study.

#### 3.3.3 Functional characterization of DE genes

Having assessed the overall consistency of the transcriptional profiles across cohorts, to further characterize the transcriptomic landscape associated with HCC and identify the biological processes altered in tumor samples, Gene Ontology (GO) enrichment analyses were performed separately on upregulated and downregulated DE gene sets. Heatmap visualization based on DE gene expression divided genes according to their modulation pattern, forming a cluster of downregulated genes (cluster 1, orange) and one including upregulated genes (cluster 2, green) (Fig. 3, panel a), consistent with DE analysis results. GO enrichment analysis revealed a marked imbalance between downregulated and upregulated biological processes. Specifically, the most significantly enriched GO terms were predominantly associated with genes downregulated in HCC and retained strong statistical significance after multiple testing correction, whereas upregulated processes showed weaker and more heterogeneous enrichment profiles (Fig. 3, panel b). Among the top downregulated biological processes, terms related to regulation of translation and cytoplasmic translation were strongly enriched (43/201 and 12/26 genes, respectively; adjusted p ≤ 5.5 × 10^−4^), suggesting a broad suppression of translational control mechanisms in tumor tissues. This transcriptional signature is consistent with dedifferentiation and chronic stress–induced translational shutdown. Fatty acid oxidation was also significantly downregulated (17/51 genes; adjusted p ≈ 5.5 × 10^−4^), indicating altered hepatic oxidative metabolism and mitochondrial impairment. Similarly, enrichment of the macromolecule biosynthetic process term (37/189 genes; adjusted p ≈ 8.8 × 10^−4^) supported a broader reduction in anabolic and biosynthetic functions. Notably, the epoxygenase cytochrome P450 pathway was significantly downregulated (9/16 genes; adjusted p ≈ 1.0 × 10^−3^), consistent with impaired xenobiotic metabolism and loss of specialized hepatic functions typically associated with differentiated hepatocytes. In contrast, the top upregulated GO biological processes in HCC included terms related to immune regulation, cell cycle–associated mechanisms, and ribosomal RNA turnover. However, these enrichments were characterized by small gene overlaps and did not reach statistical significance after multiple testing correction (adjusted p ≈ 1), indicating weaker and more heterogeneous transcriptional activation patterns, potentially reflecting differences in cellular composition and tumor microenvironment.

**Fig. 3:**
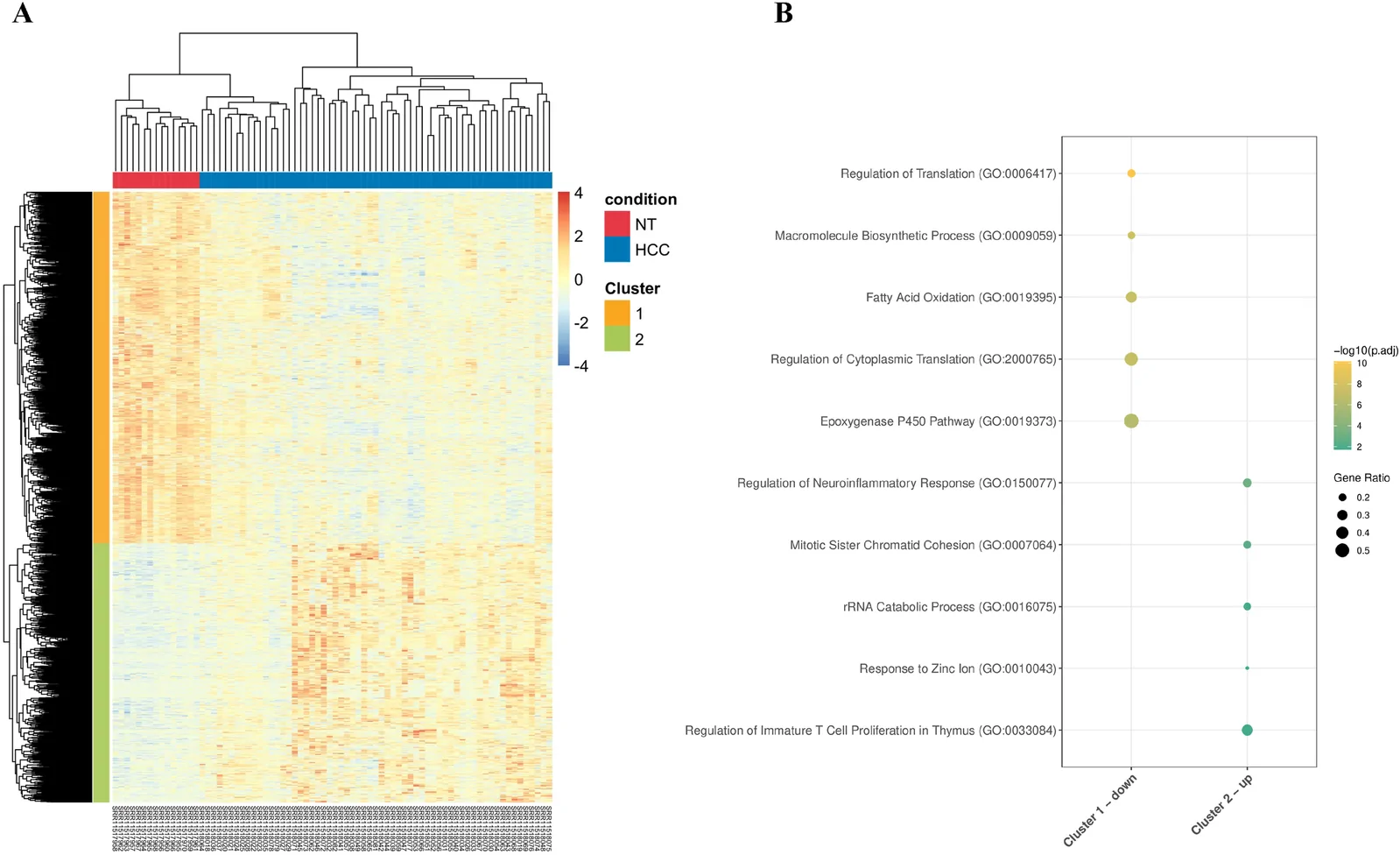
**Enriched cellular processes in HCC samples.** Panel a) Heatmap of DE genes' expression. Column dendrogram represents patients' clustering (red = NT samples; blue = HCC samples) and row dendrogram the genes' clustering based on their modulation (DEL- = 1, green; DEL+ = 2, orange). Panel b) Gene Enrichment dot plot of the 5 most enriched pathways among GO Biological Process 2025 library [31] according to a p-adj ≤ 0.01 for DEL- (Cluster 1) and DEL+ (Cluster 2) genes. Dot color represents -log10(p-adj) values, and dot size represents the ratio between the number of DE genes from the biological process and the total number of genes belonging to that process.

### 3.4 Evaluation of covariates influencing HERV transcriptional variability

In addition to the overall DE HERV analysis, to evaluate the potential contribution of clinical variables to HERV transcriptional variance, exploratory analyses were performed considering gender, viral etiology, and tumor histological grade. Given the uneven distribution of samples across etiological and grade groups, these analyses were intended to evaluate broad sources of transcriptional variability rather than to establish subgroup-specific HERV signatures.

#### 3.4.1 Impact of clinical and biological covariates on HERV transcriptional variance

Pairwise PCA plots confirmed that the partial separation between HCC and NT samples was mainly associated with PC1, explaining 14.6% of the total variance (Supplementary Fig. S4a). Among the evaluated covariates, gender showed a partial association with sample distribution along PC3, accounting for approximately 5% of the variance (Supplementary Fig. S4b). In contrast, neither viral etiology nor tumor grading showed evident contributions to the overall clustering patterns observed in the PCA space (Supplementary Fig. S4c and d). However, the limited and uneven representation of the different etiological and grade categories, particularly the small HCV-associated subgroup, precludes exclusion of more subtle subgroup-specific effects.

To further evaluate the impact of these covariates on HERV modulation, the differential expression analysis between HCC and NT samples was repeated including each covariate individually in the experimental design while maintaining tumor status as the primary variable of interest. Consistent with the PCA results, most DE HERVs identified in the covariate-adjusted analyses overlapped with those detected in the original analysis, further supporting HCC presence as the major factor driving HERV transcriptional modulation (Supplementary Fig. S4e). Among the evaluated variables, gender adjustment identified 131 DE HERVs, including 105 upregulated and 26 downregulated loci, of which 127 overlapped with the DE HERVs identified in the original analysis. These results indicated substantial consistency between the two approaches and suggested a limited impact of gender imbalance on the major HERV transcriptional signatures identified in this cohort. Similarly, adjustment for viral etiology showed extensive overlap with the original DE analysis. Given that the etiologically characterized cohort was strongly dominated by HBV-associated tumors, while HCV-associated and non-viral cases were represented by much smaller groups, this result should not be interpreted as evidence of equivalent HERV transcriptional profiles across etiologies. Rather, within the statistical limits of this cohort, tumor status accounted for a larger component of the detected HERV transcriptional differences than the available etiological categories.

#### 3.4.2 HERV transcriptional modulation according to tumor histological grade

To evaluate whether the global HCC-associated HERV signatures varied according to histological grade, we calculated, for each sample, the mean standardized expression (Z-score) of the 145 HERV loci upregulated and the 40 loci downregulated in the overall HCC-versus-NT analysis. Both signatures showed clear grade-associated expression patterns, with the aggregate upregulated-HERV score increased across G1–G3 tumors, whereas the downregulated-HERV score showed the opposite trend (Fig. 4, panel a). Differences among G1, G2, and G3 tumors were significant for both signatures. When NT samples were included as an external reference group, Spearman rank analysis further showed a significant monotonic association across NT and increasing histological grades, with opposite directions for the upregulated and downregulated signatures (Fig. 4, panel a). These findings indicate that the magnitude of the HCC-associated HERV transcriptional dysregulation is associated with histological grade within the analyzed cohort. Because the analyzed samples are cross-sectional, these patterns should, however, not be interpreted as longitudinal activation or silencing of HERVs during tumor progression.

**Fig. 4:**
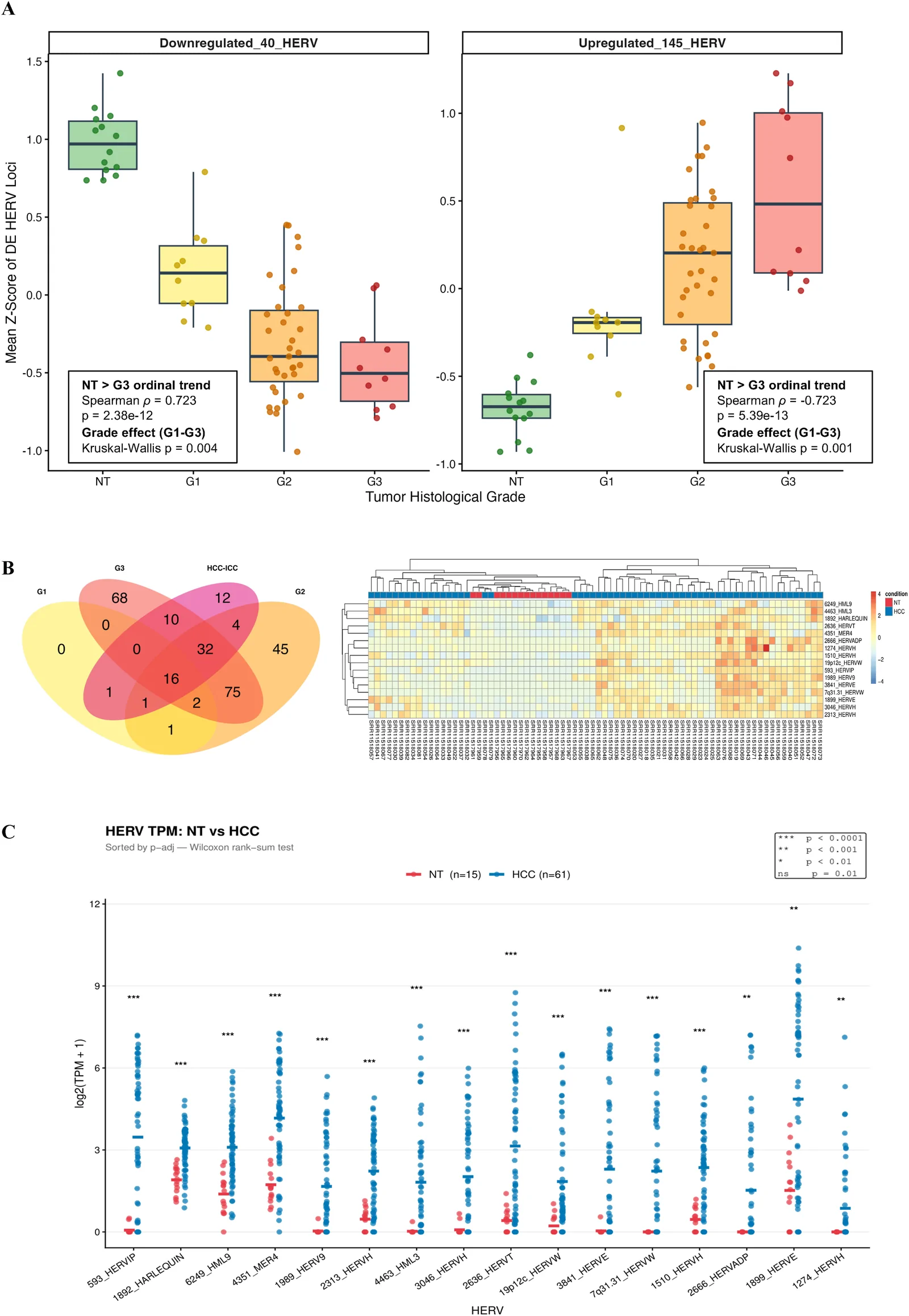
**Analysis of HERV modulation according to HCC grade.** Panel A: Grade-associated expression patterns of HCC differentially expressed HERVs. Boxplots show the distribution of aggregate HERV signature mean Z-scores across non-tumoral controls (NT) and HCC samples stratified by histological grade (G1–G3); individual dots represent single samples. Differences among G1, G2, and G3 tumors were assessed using the Kruskal–Wallis test, while monotonic trends across the ordinal NT–G1–G2–G3 groups were evaluated using Spearman rank correlation. Panel B: Quadruple Venn diagram showing the DE HERVs specifically modulated in a specific HCC grade and shared between two or more grades (left) and heatmap based on the variance of expression of the 16 DE HERVs upregulated in all tumor histological grades as compared to NT. Panel C: Expression levels of the 16 common DE HERVs upregulated in all HCC grades. Significance of differences between groups was assessed using the Wilcoxon rank-sum test, and significance levels are indicated by asterisks (*p < 0.01; **p < 0.001; ***p < 0.0001).

To further investigate HERV modulation across the HCC cohort, dedicated DE analyses have been performed according to tumor histological grading (G1, G2, G3) and ICC comorbidity (HCC-ICC), by individually comparing each subgroup to NT samples. The resulting DE HERV sets have then been compared to identify shared and subgroup-specific transcriptional signatures (Fig. 4, panel b). The HCC G1 subgroup showed the lowest number of DE HERVs (n = 21), none of which were uniquely identified in this grade. Specifically, all DE HERVs identified in G1 were shared with at least one additional subgroup. In contrast, larger numbers of DE HERVs were identified in G2 and G3 tumors. The G2 subgroup showed 176 DE HERVs, including 45 specifically modulated loci, whereas G3 tumors presented the highest number of DE HERVs overall (204), including 69 uniquely modulated elements. These findings indicate that the extent of HERV transcriptional deregulation differed across histological grades within the analyzed cohort, with the largest number of DE loci observed in G3 tumors. The HCC-ICC subgroup displayed 76 DE HERVs, including 12 loci uniquely associated with this category, potentially reflecting transcriptional features related to ICC comorbidity. Notably, 16 DE HERVs were consistently shared among all HCC grades and showed concordant upregulation in all conditions (Fig. 4, panel b).

To further characterize the transcriptional behavior of the DE HERVs in common or specific to each HCC subgroup, heatmaps based on their variance of expression were generated. The heatmap based on the 16 upregulated HERVs shared across all HCC histological grades showed a partial segregation of HCC and NT samples, with a subset of HCC samples displaying markedly increased HERV expression relative to both NT tissues and the remaining HCC cohort (Fig. 4, panel b). These HCC samples form a clearly separated group and are further divided into two subclusters according to increasing HERV expression. Comparable clustering patterns were observed for the DE HERVs associated with G2 and G3 subclasses, where tumor samples generally exhibited higher and more homogeneous HERV expression profiles compared with NT tissues (Supplementary Fig. S5). Importantly, histological grade itself did not determine the major sample subclusters, consistent with the heterogeneous transcriptional profiles observed within individual grades. Despite considerable inter-individual variability, all 16 HERV loci showed significantly higher TPM values in HCC tissues (mean TPM between 5.6 and 170.3) in contrast with a basal or absent expression in NT samples, with p-values < 0.001 (13 out of 16) or < 0.01 (3 out of 16), supporting the consistency of their differential expression within the analyzed cohort (Fig. 4, panel c)

To better characterize the grade-associated expression patterns of the 16 HERV loci consistently upregulated across G1–G3 tumors, we calculated their aggregate standardized expression score in each sample. The 16-HERV signature showed a significant positive monotonic association across NT and increasing histological grades (Spearman ρ = 0.698, p = 1.91 × 10^−11^), while expression also differed significantly among G1–G3 tumors (Kruskal–Wallis p = 0.013), indicating a grade-associated increase in the magnitude of this shared HERV transcriptional signature (Fig. 5, panel a). We next performed an exploratory ROC analysis to prioritize individual loci according to their discriminatory performance between G1 HCC and NT tissues within the discovery cohort. Among the 16 loci, 2636_HERVT, 4351_MER4, 1510_HERVH, and 1892_Harlequin showed the highest AUC values (1.000, 0.993, 0.967, and 0.933, respectively) (Fig. 5, panel b). These loci also discriminated the overall HCC cohort from NT samples, individually and when considered jointly (Fig. 5, panel c). Because candidate selection and ROC evaluation were performed within the same discovery cohort, these estimates should be regarded as exploratory and require validation in independent samples before any diagnostic relevance can be established.

**Fig. 5:**
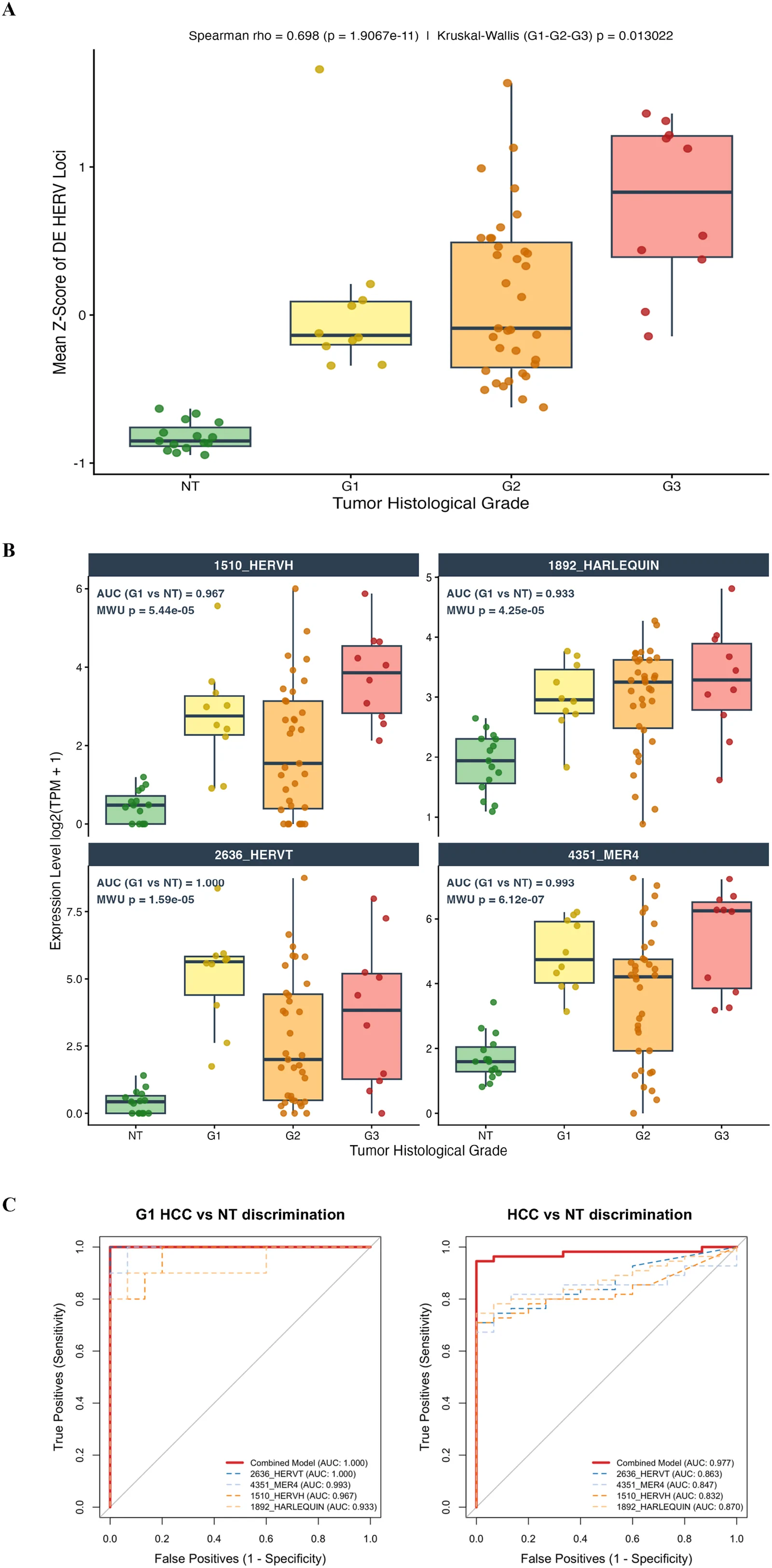
**Grade-associated expression and exploratory performance of the 16 HERV loci consistently upregulated across HCC histological grades** Panel B: Candidate HERV loci discriminating G1 HCC from NT tissues. For each sample, the mean standardized expression (Z-score) across the 16 loci was calculated. Differences among G1–G3 tumors were assessed using the Kruskal–Wallis test, while monotonic trends across NT and increasing histological grades were evaluated using Spearman rank correlation. Panel B: Expression profiles of the four HERV loci showing the highest discriminatory performance between G1 HCC and NT tissues. Panel C: Receiver operating characteristic (ROC) curves and area under the curve (AUC) values for the four selected HERV loci, evaluated individually and in combination for discrimination of G1 HCC from NT tissues and of the overall HCC cohort from NT controls.

### 3.5 Functional evaluation of the 16 upregulated HERVs shared across HCC subclasses

Given the consistent upregulation of 16 HERV loci across G1–G3 HCC histological grades within the analyzed cohort, these elements were further investigated to infer their transcriptional features, including genomic context of integration, transcript architecture, and residual coding potential. These analyses were intended to prioritize candidate HERV-associated transcriptional events for subsequent independent experimental validation.

#### 3.5.1 HERV-associated pathway enrichment across HCC grades

To investigate the biological pathways associated with the 16 HERV loci consistently upregulated across G1–G3 HCC samples, gene set enrichment analyses (FGSEA) were performed using cellular genes correlated with each HERV locus (Fig. 6). The resulting enrichment profiles revealed marked heterogeneity and grade-associated differences among HERV loci. In G1 tumors, most HERV loci were associated with negative enrichment of metabolic and inflammatory pathways, including hypoxia, glycolysis, IL6/JAK/STAT3 signaling, IL2/STAT5 signaling, coagulation, oxidative phosphorylation, as well as fatty acid, bile acid, and xenobiotic metabolisms. For example, a strong negative enrichment of bile acid metabolism was observed for 1274_HERVH, 1899_HERVE, 3841_HERVE, and 6249_HML9. In contrast, pathways related to cell proliferation and cell-cycle progression - such as E2F targets, G2M checkpoint signaling, and mitotic spindle organization - showed positive enrichment for a subset of HERV loci. A marked shift in enrichment profiles was observed in G2 tumors. Several HERV loci that were negatively associated with metabolic and stress-response pathways in G1 became positively associated in G2. For example, 2666_HERVADP, 1274_HERVH, and 2313_HERVH displayed positive enrichment for fatty acid metabolism, cholesterol homeostasis, bile acid metabolism, and oxidative phosphorylation pathways. Similarly, 2636_HERVT and 593_HERVIP shifted from negative to positive association with stress-response pathways, including mTORC1 signaling, PI3K/AKT/mTOR signaling, and unfolded protein response pathways. Overall, these results indicate HERV-associated correlation patterns between G1 and G2 tumors, deserving further investigation to assess a possible functional transition between histological grades. In G3 tumors, several HERV loci showed enhanced positive enrichment for inflammatory and interferon-related pathways and/or proliferation-associated programs, whereas a subset of HERVs maintained a stable negative association with metabolic pathways across all histological grades. Lastly, the HCC-ICC subgroup exhibited a distinct enrichment profile compared to G1-G3 grades. Several HERV loci showed strong positive association with the cell proliferation-related pathways, whereas immune-related and metabolic pathways displayed greater heterogeneity. Overall, these results indicate that while some HERV loci maintain relatively stable enrichment profiles across HCC histological grades, others show marked grade-associated differences in their transcriptional correlations with metabolic, inflammatory, stress-response, and proliferative pathways.

**Fig. 6:**
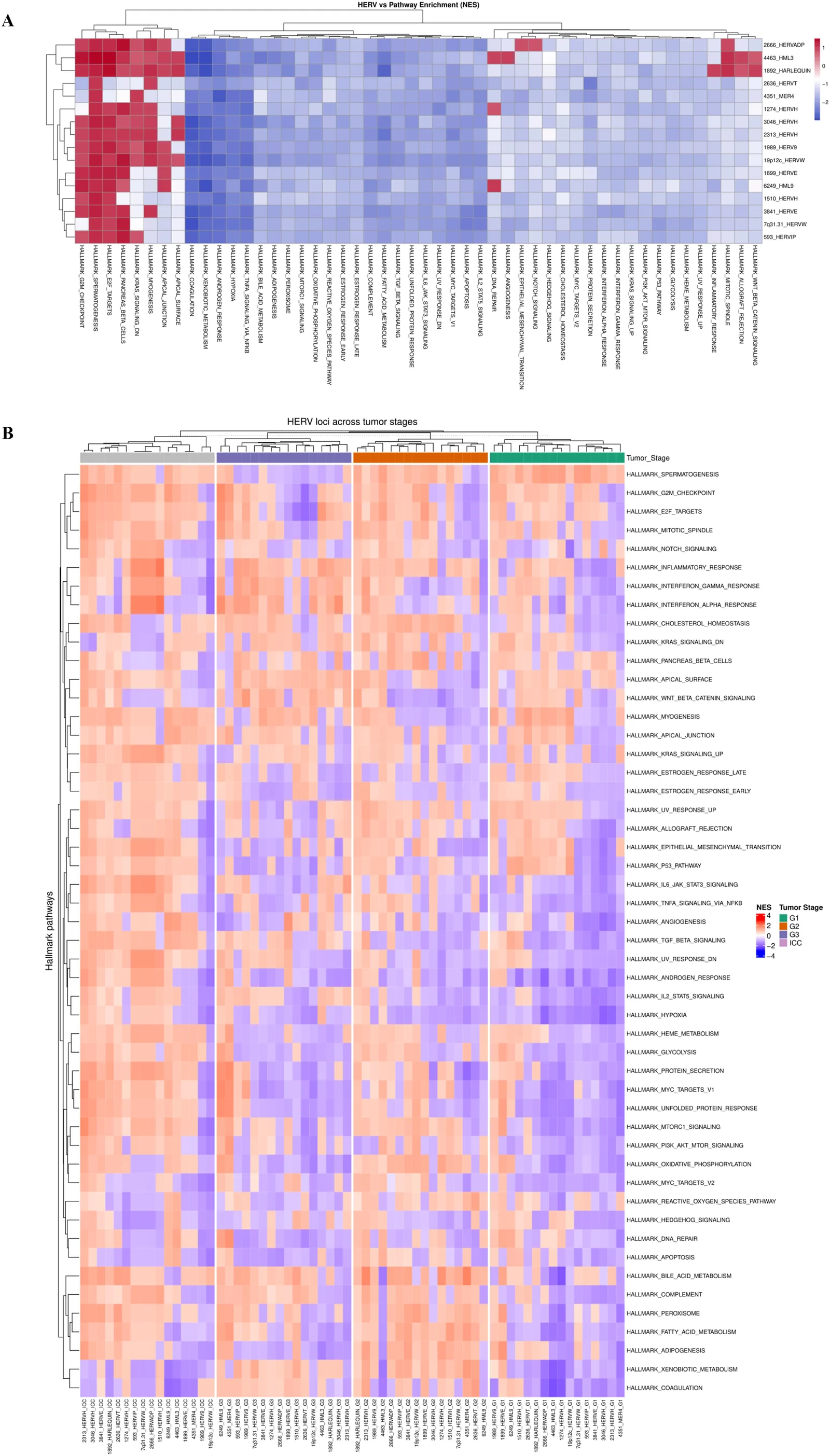
**FGSEA-based pathway enrichment of Msigdb hallmark pathways associated with 16 HERV loci across HCC tumor grades.** Genes were ranked according to Spearman's correlation between HERV loci and gene TPM matrix, and pathway enrichment scores were calculated using FGSEA. (a) Heatmap showing normalized enrichment scores (NES) for pathways enriched for 16 HERV loci. (b) Heatmap showing NES stratified by tumor grades (G1, G2, G3, and ICC comorbidity group).

#### 3.5.2 De novo reconstruction and characterization of HERV-derived transcripts

To further characterize the 16 DE HERV loci upregulated across all analyzed HCC histological grades, their genomic localization was examined with respect to overlapping cellular genes (Table 2). Although these analyses cannot establish biological or regulatory function, they were used to prioritize HERV loci with transcriptional and genomic features warranting further investigation.

**Table 2:** **Characterization of the 16 common DE HERVs upregulated in all HCC histological grades**. The HERV TPM is expressed as arithmetic mean ± the corresponding standard deviation in NT and HCC samples, HERV colocalization with cellular genes is reported, indicating whether the HERV is integrated within exons and/or introns and the relative exons/introns total number. NE = not expressed. * = reported as untranslated exon.

DEL + HERVs COMMON TO ALL GRADES			COLOCALIZED GENE				INTEGRATION	
HERV ID	TPM		Name	Coding	DE	Reported in HCC	exon	intron
	NT	HCC						
7q31.31_HERVW(+)	NE	20,4 ± 38,8	LINC02476(−)	no	+	[40], [41], [42], [43]	0	1
2666_HERVADP(−)	NE	13,9 ± 34,9	intergenic					
1274_HERVH(−)	NE	4,5 ± 18,5	intergenic					
593_HERVIP(−)	0,0 ± 0,1	32,7 ± 39,5	intergenic					
2636_HERVT(+)	0,4 ± 0,5	38,3 ± 79,3	AKR1B15(+)	yes	+	[44]	2*	2
1989_HERV9(+)	0,0 ± 0,1	5,6 ± 9,4	intergenic					
4351_MER4(+)	2,8 ± 2,3	34,1 ± 38,1	ACSM1(−)	yes	no	[41], [45]	0	1
3841_HERVE(+)	0,0 ± 0,1	23,3 ± 44,0	LINC02882(−)	no	+	[46]	0	1
4463_HML3(−)	0,0 ± 0,1	12,6 ± 31,9	B4GALNT2(+)	yes	NE	[47], [48], [49]	1*	0
3046_HERVH(−)	0,1 ± 0,2	8,4 ± 13,2	intergenic					
1510_HERVH(+)	0,4 ± 0,4	9,3 ± 14,4	ENSG00000306929.1(−)	no	NE		1*	1
2313_HERVH(−)	0,4 ± 0,4	6,3 ± 6,6	ENSG00000286540.2(+)	no	no		0	1
19p12c_HERVW(+)	0,2 ± 0,3	10,5 ± 21,8	no gene					
1892_Harlequin(−)	2,9 ± 1,3	8,7 ± 5,2	RPS23(−)	yes	no	[50], [51]	1*	0
1899_HERVE(+)	3,3 ± 4,1	170,3 ± 261,6	intergenic					
6249_HML9(+)	1,9 ± 1,4	11,7 ± 12,5	ENSG00000286747.2(+)	no	+		2*	1
			LINC02897(−)	no	no		4*	2

The 16 DE HERVs present a complete proviral structure, except for 4351_MER4 (chr 16), which is interrupted by several secondary integrations (Alu and L1 elements). In addition, 7q31.31_HERVW (chr 7) 6249_HML9 (chr 1), and 3046_HERVH (chr 9) lacked either the 5′ or the 3’ LTR. Nine of 16 HERV loci were colocalized with a total of 10 cellular genes, of which four were protein coding while six were annotated as long non-coding RNAs (lncRNAs) (Table 2). Among these overlapping genes, four were also DEL+ in the present study, and six have been previously investigated for their probable involvement in HCC and other tumors. Regarding the relative localization with respect to the gene, three of the four DE HERVs found in protein-coding genes are integrated within untranslated regions (UTR), showing the same orientation in two cases. This genomic configuration is compatible with the possibility that HERV sequences may be incorporated into gene-associated transcripts. Likewise, antisense overlap may theoretically permit the generation of complementary dsRNA transcripts and consequent gene-silencing. However, genomic overlap and orientation alone do not demonstrate a functional interaction, which thus cannot be inferred from the present data. To predict the transcriptional architecture of these loci, we performed the *de novo* reconstruction of their transcripts from HCC and NT transcriptomes. Of note, the great majority of the reconstructed transcripts were inferred exclusively in HCC, consistent with the locus-level differential expression and TPM analyses. Among the 16 upregulated HERVs, we focused our attention on 2636_HERVT and 4463_HML3, since the related transcripts exhibited relatively high TPM values and concordant direction as the corresponding proviral sequence and/or were colocalized with genes having a proposed role in HCC or tumorigenesis in general (Table 2).

2636_HERVT(+) on chromosome 7 overlaps in sense orientation with the protein-coding gene AKR1B15(+), also upregulated and under investigation for its involvement in HCC [38] (Fig. 7). The HERV 5′LTR is located immediately upstream of the gene 5′UTR and includes a putative proximal enhancer site according to ENCODE Candidate Cis-Regulatory Elements (cCREs) annotations. The first two AKR1B15 exons (both annotated as UTRs) overlap the HERV internal portion (Table 2), and transcriptome reconstruction predicted transcripts spanning HERV and AKR1B15 sequences in both sense and antisense orientations(Fig. 7). In particular, transcript isoform DN454_c0_g1_i1(+) (1) showed 100% identity with reported mRNAs EF539862 and EF539865 – annotated as HERVT putative reverse transcriptase (*pol*) pseudogene - and AB167319 – reported as HERV HC2 *pol* transcript (where HERV HC2 is closely related to ERVS71, i.e. the HERVT internal reference sequence) [52]. Alignment of the reconstructed transcript with ERVS71 showed that DN454_c0_g1_i1 includes the terminal portion of *gag* as well as the *pol* and *env* genes. To evaluate its residual protein-coding potential, the reconstructed transcript was translated into all three forward frames and compared to the HERVT Gag, Pro, Pol, and Env reference protein sequences of Dfam, detecting multiple stop codons, frameshifts, and deletions.

**Fig. 7:**
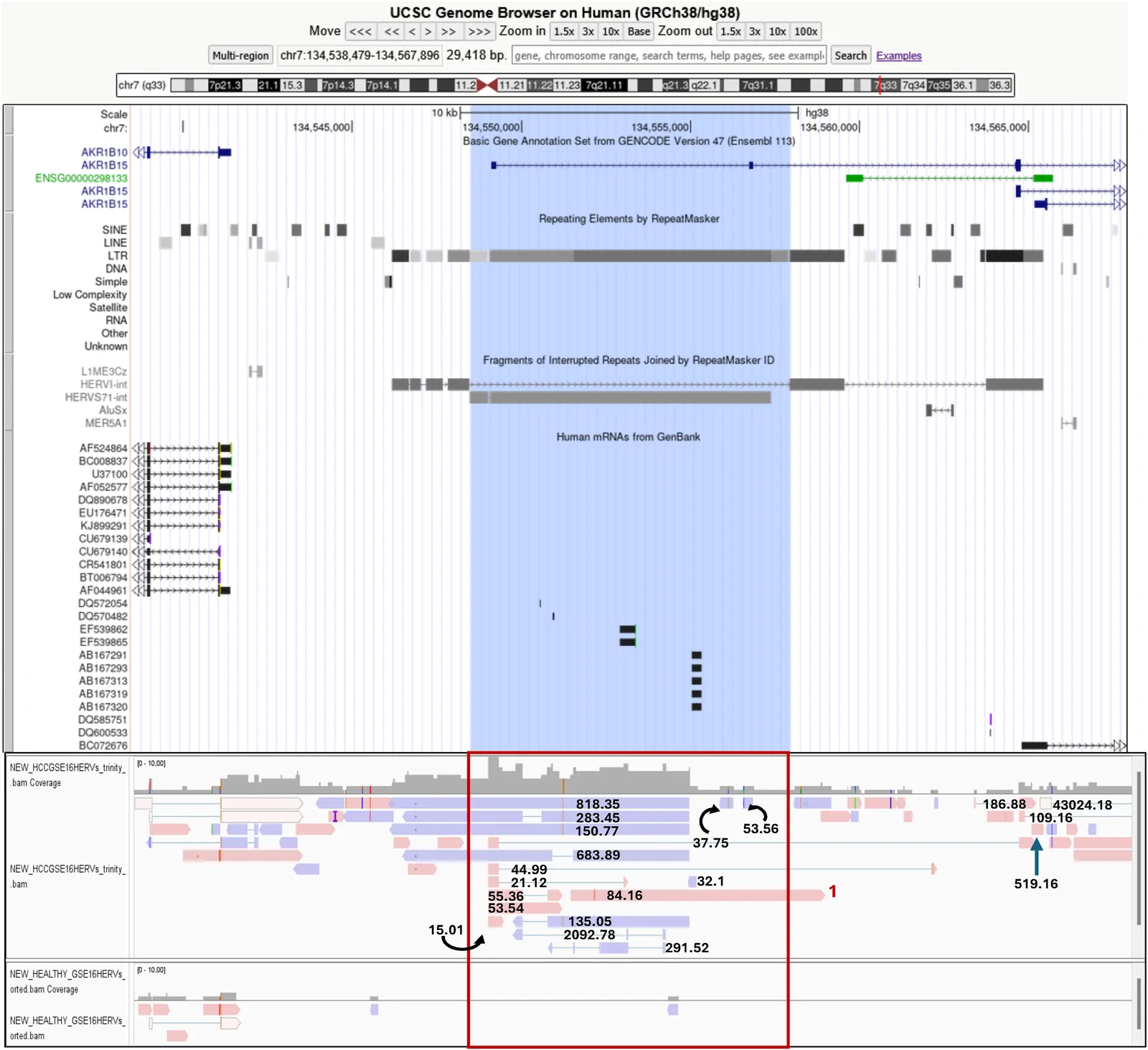
**2636_HERVT genomic environment and transcriptional activity** Panel showing 2636_HERVT locus (+10 kb at 5′ and 3′) from USCC Genome Browser Gencode version 47 (above) and IGV software (below). Genome Browser overview reports the HERV locus highlighted in blue, the colocalized gene AKR1B15(+) (DEL+ in HCC samples, HCC TPM = 1.56 ± 2.9, NT TPM = 0.01 ± 0.04) and human mRNAs EF539862 (+), EF539865(+), AB167291(+), AB167293(+), AB167313(+), AB167319(+), AB167320(+). IGV overview reports the transcript production in HCC and NT samples. In sense directed transcripts (5’ > 3′) = red; reverse directed transcripts (3’ > 5′) = blue; darker color = mapping high quality; lighter color = mapping low quality.

4463_HML3(−) in chromosome 17 is colocalized with B4GALNT2(+) protein-coding gene, not DE in our analysis and under investigation for its role in HCC [48]. The HERV overlaps with an extended 3′UTR present only in one transcript isoform (ENST00000393354.7), possibly consistent with a readthrough mechanism, although the underlying transcriptional event cannot be established from the present RNA-seq data alone (Fig. 8). Accordingly, the reconstructed HCC transcript DN491_c2_g1_i3(+) (1) is produced by B4GALNT2 and includes the HERV locus in its terminal region, showing near complete identity with ENST00000393354.7 and the reported human mRNA AK027110(+) [53]. As above, the transcript translation showed the occurrence of multiple stop codons, frameshifts, and deletions, suggesting the loss of protein-coding potential.

**Fig. 8:**
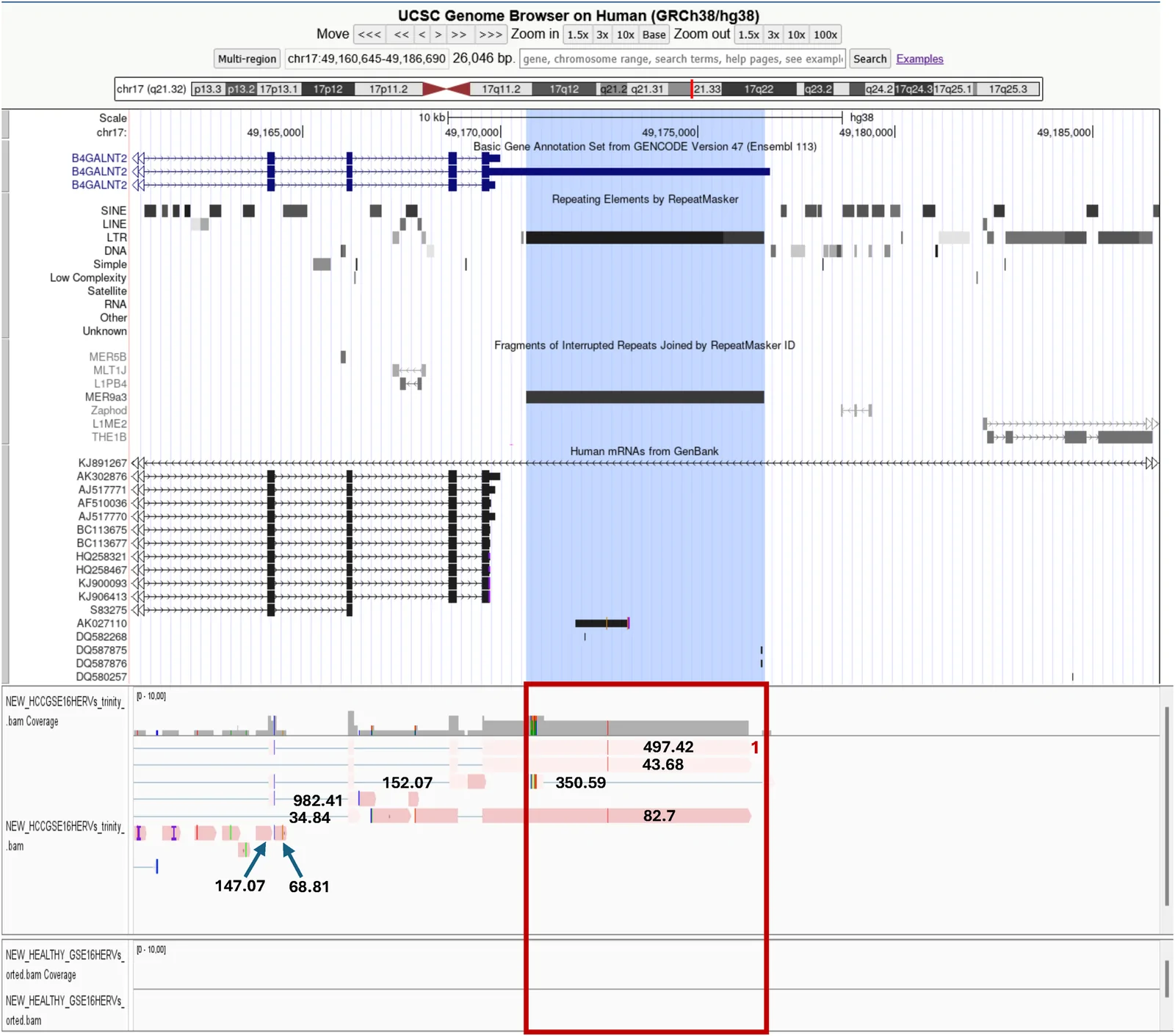
**4463_HML3 genomic environment and transcriptional activity** Panel showing 4463_HML3 locus (+10 kb at 5′ and 3′ ends) from USCC Genome Browser Gencode version 47 (above) and IGV software (below). Genome Browser overview reports the HERV locus highlighted in blue, the colocalized gene B4GALNT2(+) (NA in HCC samples, HCC TPM = 0.02 ± 0.06, NT TPM = 0 ± 0) and human mRNA AK027110(+). IGV overview reports the transcript production in HCC and NT samples. In sense directed transcripts (5’ > 3′) = red; reverse directed transcripts (3’ > 5′) = blue; darker color = mapping high quality; lighter color = mapping low quality.

## 4 Discussion

HCC is among the leading causes of cancer-related deaths worldwide [1], [2], also due to the limited availability of validated biomarkers and disease-specific therapeutic targets. In this frame, HERVs are emerging as promising candidates, since i) their expression is altered in this and other human cancers [8], [9], [10], and ii) chronic hepatitis caused by HBV and HCV, i.e. the first risk factors of HCC, have been shown to modulate HERV activation [8], [14], [21], [22]. The present study focused on the locus-resolved analysis of HERV transcriptional modulation in a bulk RNA-Seq dataset of liver tissues from HCC patients and NT controls retrieved from the GEO Databank (accession number GSE148355, [23]). In general, a major advantage of locus-resolved RNA-seq analysis is the possibility of assigning transcriptional signals to specific HERV integrations based on their unambiguous positions across the genome, thereby overcoming the limitations posed by sequence identity among repetitive elements. This highly specific approach complements targeted primer- or probe-based assays, which generally interrogate selected HERV families or loci, by enabling a broader and more detailed assessment of locus-specific transcriptional activity [54]. Dedicated computational tools such as Telescope and HERV-Fcount have been developed and benchmarked for this purpose, and locus-specific RNA-seq strategies have increasingly been applied to HERV profiling in cancer, including HCC [55], [56], [57], [58]. In this work, we relied on a curated collection of ∼3300 HERV integrations, characterized in detail for their level of integrity and coding potential [18], [27], [59], [60], [61], [62], and analyzed their individual modulation in HCC. Despite the clinical heterogeneity of the cohort, HCC condition emerged as the major factor associated with HERV transcriptional variability. Unsupervised clustering analysess highlighted the tendency of the samples to distribute according to HCC presence, and the DE analyses confirmed widespread HERV dysregulation in HCC samples, with a clear predominance of upregulated loci, together with extensive modulation of cellular genes involved in metabolic, translational, and inflammatory pathways. More in detail, the transcriptional landscape of HCC liver tissues revealed a majority of downregulated genes, in line with the predominant downregulation of pathways associated with hepatocyte differentiation and metabolic homeostasis. Among the most significant repressed human gene elements, RMPM, VDBP, ERRFI1, and AMBP were reported to be downregulated also in other HCC studies, with the last two being investigated as possible prognostic markers [63], [64], [65], [66], while ITIH2 and RNF152 have been associated with longer overall survival [67] and lower HCC progression [68], respectively, hinting at their negative regulation role in HCC surveillance. According to our gene enrichment analysis, ERRFI1 was included among 18 GO processes, with the most significant being the regulation of epidermal cell differentiation (GO:0045604). Such a process is under investigation for its role in favoring HCC growth, as ERRFI1 negative regulation in the tumor is associated in turn with EGFR (epidermal growth factor receptor) increase [63], [69]. AMBP, a bikunin precursor expressed predominantly in liver parenchyma, was represented in 17 enriched processes, with the most significant being the negative regulation response to stimulus (GO:0048585). Previous studies suggested the involvement of AMBP expression in anticancer mechanisms that are mainly inhibited in HCC, such as cellular invasion and metastasis inhibition, hinting that the gene's downregulation might sustain tumor progression [64]. More in general, when considering the top 5 GO processes associated with downregulated genes, the enrichment analysis in HCC highlights a dominant transcriptional signature characterized by the concerted downregulation of hepatocyte-specific metabolic, biosynthetic, and translational programs, including fatty acid oxidation, xenobiotic metabolism, translational regulation, and cytochrome P450-associated functions. These findings are consistent with the extensive metabolic rewiring and progressive loss of specialized hepatic functions typically observed during hepatocarcinogenesis, particularly in the presence of chronic viral liver injury [70], [71], [72].

Within this context, HERV modulation appears integrated into the broader transcriptional remodeling associated with HCC rather than representing an isolated phenomenon. DE analysis identified a total of 185 modulated HERV loci (∼6% of the analyzed elements), with a preponderant upregulation in HCC (n = 145). The predominance of virally associated tumors in the cohort, particularly HBV-associated HCC, does not allow a direct causal relationship between viral infection and HERV activation to be established, but still provides a relevant biological context. Viral infections are known to influence host transcription, inflammatory signaling, and epigenetic regulation, all of which may sustain oncogenic transformation and provide conditions permissive to altered HERV expression. Human T-Cell leukemia virus-1 (HTLV-1), the cause of adult T-cell leukemia and HTLV-1-associated myelopathy, has been associated with activation of HERV-W, HERV-H, HERV-K(HML2), and HERV-E LTRs and with the prevalence of antibodies against HERV-K10 Pol and Gag peptides [14], [73], [74]. Kaposi's Sarcoma-Associated Herpesvirus (KSHV), able to induce Kaposi's sarcoma and primary effusion lymphoma, has been linked to HERV-K(HML2) transactivation [14], [75]. Epstein-Barr Virus (EBV), correlated to Burkitt's lymphoma, Hodgkin's lymphoma, nasopharyngeal cancer, NK/T-cell lymphoma, and post-transplant lymphoma, has been related to HERV-K18 activation [14], [76]. Concerning viral hepatitis, previous studies reported the modulation of the Syncytin-1 locus (7q21.2_HERV-W) in the presence of HBV, and the general expression of HERV-H and HERV-K(HML2) groups in HCV-positive individuals [8], [14], [21], [22]. Compared with previous investigations conducted mainly at the whole group expression level, our locus-resolved analysis identified specific modulated members from the betaretrovirus-like HERV-K supergroup (HML2, HML3, HML6) and gammaretrovirus-like HERV-W, HERV-H, and HERV-E groups, in line with previous reports describing their general deregulation in HCC [8], [13], [77]. In more detail, we identified 34 HERV-H loci (22 DEL+), 14 HERV-W elements (9 DEL+), and 30 HERV-K supergroup members (27 DEL+), with HML3 as the most abundant group, comprising 9 DE loci (of which 8 DEL+). Some recent works have substantially expanded the characterization of HERV transcription in HCC using locus-resolved RNA-seq approaches on different HCC cohorts. The main characteristics of these studies – including cohort composition, RNA-seq design, and results in terms of DE HERVs identified and their validation for prognostic and molecular classification and/or in vitro effects – are summarized in Supplementary Table S2. Regarding total RNA-seq profiles, i.e. the same layout of our dataset, Chang et al. analyzed a Taiwanese HCC cohort using Telescope and identified survival-associated HERV loci, HERV-based patient subgroups, nearby gene associations, and immune- and metabolism-related transcriptional programs [58]. More recently, HERV-Fcount was applied to the same cohort to identify a set of 14 survival-related HERV loci and to assess their transcriptional changes following perturbation in HCC cell lines [56]. Two other studies were conducted on poly(A)RNA-seq data from TCGA-LIHC cohort: Wen and coauthors performed a locus-specific analysis and identified 216 DE HERV loci, with a predominance of downregulated elements (n = 166) [78]; while Igarashi et al. focused on a restricted set of HERV elements epigenetically suppressed by SETDB1 and experimentally linked their derepression to viral-mimicry-associated responses [57]. In general, the prevalent HERV upregulation that we observed in our study is in line with the results obtained with the Taiwanese CMUH cohorts described by Chang and colleagues [56], [58], while both studies are in contrast to the global HERV suppression patterns reported in the TCGA-LIHC cohort [78]. Rather than indicating inconsistency, these differences highlight the strong dependence of locus-specific HERV profiles on RNA-seq design. Unlike the poly(A) capture methods utilized in TCGA-LIHC, which inherently bias the analysis towards the minority of HERV transcripts with 3′ polyadenylation, total RNA-seq captures the full breadth of RNA species, including the substantial fraction of non-coding ones. Another source of discrepancy in DE HERV profiles is likely represented by cohort composition: in our case, HCC cases showed a predominant viral etiology (69%), and NT samples derived from different individuals affected by non-HCC liver diseases (cholangiocarcinoma and polycystic liver disease) in the absence of infection [24]. Differently, both the Taiwanese and TCGA-LIHC cohorts included a minority of uninfected individuals (43% and 9%), with control samples represented by paired adjacent NT tissues. Finally, the bioinformatic pipeline also presented relevant differences in the reference dataset used to count reads mapped at HERV loci and the statistical thresholds adopted for DE analysis. The locus-level comparison with previous results based on the overlap of DE-HERV genomic coordinates showed the presence of 6 common DE HERV loci (4463_HML3, 6249_HML9, 6072_HERV4, 4682_HERV3, 2003_HERV9, and 12q24.12_HML6) in the case of Wen et al. [78], all having an opposite modulation between the two studies. Considering the study of Chang and colleagues [56], two other DE-HERVs were shared with our results, showing in this case concordant upregulation: 1989_HERV9 and 491_HERVH. Interestingly, both HERV loci were included in the set of 14 survival-related DE HERVs, whose overexpression in HCC has been significantly correlated with poor overall survival, and reported to act as positive regulators by the coordinated expression in the same direction as their closest downstream long non-coding RNAs [56].

Overall, our results are in agreement with other available reports about HERV modulation in HCC [8], [10], [11], [56], [58] and suggest that chronic inflammatory signaling, oxidative stress, and interferon-mediated responses associated with viral hepatitis may create permissive conditions for HERV transcriptional activation.

We next examined HERV transcriptional patterns according to histological grade, stratifying samples from well-differentiated HCC (G1) – characterized by low proliferative capacity, low aggressiveness, and better prognosis – to intermediate and poorly differentiated HCC (G2 and G3, respectively) [39], [79]. G1 tumors displayed the lowest number of DE HERVs and lacked uniquely modulated loci, whereas G2 and G3 tumors showed progressively larger numbers of grade-associated DE HERVs. Because these groups derive from a cross-sectional cohort and contain relatively limited sample numbers, this pattern cannot be interpreted as evidence of a longitudinal increase in HERV expression during tumor progression. Nevertheless, the additional signature-level analysis provided independent support for an association between HERV transcriptional dysregulation and histological grade. When DE HERVs identified as upregulated or downregulated in the overall HCC-versus-NT comparison were considered as an aggregate transcriptional signature, their mean standardized expression increased or decreased across G1–G3 tumors, respectively. Significant differences among G1, G2, and G3 tumors were observed for both signatures, while inclusion of NT samples as an external reference revealed significant monotonic trends across NT and increasing histological grades. Thus, the grade-associated pattern was not limited to differences in the number of DE loci identified in each subgroup but was also reflected in the magnitude of expression of the global HCC-associated HERV signatures at the individual-sample level. These findings indicate that the extent of HERV transcriptional deregulation differed across histological grades within the analyzed cohort, and is greater, on average, in less differentiated tumors, potentially reflecting increasing epigenetic instability and transcriptional remodeling.

Among the DE HERVs identified, 16 loci were consistently upregulated across all HCC histological grades and colocalized with cellular genes under investigation for their involvement in HCC and other tumors in most cases. Within the present cohort, these loci therefore represent candidate HCC-associated HERVs, whose reproducibility remains to be established in independent datasets. However, it is worth noting that 3 of these DE-HERVs (1989_HERV9, 4463_HML3, and 6249_HML9) were shared with the above locus-resolved RNA-seq studies [56], including one of the 14 DE HERVs previously associated with poor overall survival [56]. The aggregate expression of the 16 shared loci also showed a significant positive association with histological grade and differed significantly among G1–G3 tumors. To further prioritize individual members of this 16-HERV set for subsequent validation, we performed an exploratory ROC analysis within the discovery cohort. Four loci (2636_HERVT, 4351_MER4, 1510_HERVH, and 1892_Harlequin) showed the highest discriminatory performance between G1 HCC and NT tissues and retained the ability to discriminate the overall HCC cohort from NT samples both individually and in a combined model. These findings are of potential translational interest because they identify loci whose altered expression is already detectable in well-differentiated HCC. However, because both candidate selection and ROC evaluation were performed in the same cohort, the resulting AUC should be regarded solely as a means of prioritizing candidates for independent validation rather than as evidence of diagnostic performance.

Functional enrichment analyses based on cellular genes correlated with these HERVs revealed dynamic and heterogeneous associations between individual HERV loci and distinct transcriptional programs across HCC histological grades. In G1 tumors, several HERVs were predominantly associated with negative enrichment of metabolic and inflammatory pathways, whereas G2 and G3 tumors showed stronger positive association with inflammatory, interferon-related, stress-response, and proliferation-associated pathways. These findings were consistent with the previous studies describing metabolic dysfunction as an early HCC development feature [80], [81] and reporting the constitutive activation of JAK/STA3 signaling pathway in HCC [82]. Interestingly, several of these pathways shifted towards positive enrichment in G2 grade, in line with metabolic reprogramming in less differentiated tissue. Among these, PI3K/AKT/mTOR signaling has been shown to promote HCC progression by regulating glucose, lipid, and oxidative metabolism [80], [83]. Similarly, the G3 tumor grade showed a consistent positive enrichment of inflammatory and cell proliferation pathways across the 16 HERV loci. The observed shifts in pathway enrichment do not demonstrate direct regulatory interactions between HERV loci and the associated cellular pathways; however, they suggest that distinct HERV loci are embedded within different transcriptional contexts during HCC-associated transcriptomic remodeling. Interestingly, some HERV loci maintained relatively stable enrichment profiles across subclasses, while others displayed marked transitions in pathway association patterns, supporting the heterogeneous behavior of HERV transcription across tumor contexts and the need to perform locus-specific analyses.

The *de novo* transcript reconstruction analyses identified candidate HERV-associated transcriptional events in HCC tissues. Among these, reconstructed transcripts associated with 2636_HERVT and 4463_HML3 were detected predominantly in HCC samples and overlapped cellular genes previously investigated in HCC or tumorigenesis. Importantly, these observations are based on transcriptome reconstruction and genomic colocalization and do not establish that the HERV loci regulate the associated genes or exert a functional effect in HCC cells. Moreover, the reconstructed HERV-derived transcripts displayed extensive frameshifts, deletions, and premature stop codons, suggesting severe impairment of residual protein-coding potential. Despite this, residual transcriptional interaction may occur at the RNA level. For example, 4463_HML3 reconstructed transcript overlaps an extended 3′ UTR of B4GALNT2 and is therefore compatible with an extended or readthrough transcriptional architecture, i.e. a phenomenon according to which transcription continues beyond the annotated termination site [84]. A similar event has been reported for HERV-K-MEL locus – alias 16p11.2_HERVK(HML6) – whose 5′LTR overlaps with the first exon of the zinc finger protein 689 gene (ZNF689), overexpressed in HCC and involved in apoptosis suppression [18], [85]. Similarly, B4GALNT2 has been correlated with apoptosis and EMT-related proteins' expression and suggested as a promising HCC biomarker and drug target [47], [48], [49], [51]. According to GTEx-HC expression profiles, the B4GALNT2 isoform including the HERV element (ENST00000393354.7) is not expressed in the healthy liver, in line with our observation that found it exclusively in HCC samples. While there is no information about 4463_HML3 locus involvement in HCC, the same HERV element has been found deregulated in both primary and liver metastatic colorectal cancer [38], [86]. Taken together, these transcriptomic analyses prioritize 2636_HERVT and 4463_HML3 as candidate HCC-associated transcriptional events based on their locus-specific expression, genomic context, and reconstructed transcript architecture. However, their biological relevance remains to be established experimentally. Targeted RT-qPCR and long-read sequencing will be required to confirm transcript abundance and structure, while RNA-FISH or single-cell approaches could define their cellular origin. Functional perturbation studies will ultimately be necessary to determine whether these transcripts influence neighboring gene expression or other tumor-associated processes. More broadly, the 16 HERV loci identified here represent a candidate HCC-associated transcriptional signature within the analyzed cohort. To evaluate their potential as an HERV-derived biomarker panel, validation in independent cohorts will be necessary to establish its reproducibility and generalizability across clinicopathological features.

Several methodological considerations should be acknowledged when interpreting the present findings. First, the non-tumoral controls included in the analyzed cohort were not derived from healthy liver tissue but from individuals affected by cholangiocarcinoma or polycystic liver disease. To address this limitation, we compared the transcriptional signatures identified in our study with healthy liver transcriptomes from GTEx and observed overall consistency of the major HCC-associated transcriptional trends. The GTEx comparison was used as an external contextual reference rather than as part of the primary DE analysis, thereby avoiding direct integration of independently generated RNA-seq datasets with potentially substantial batch effects. Second, because bulk RNA-seq profiles reflect the combined transcriptional contribution of different cell populations, cellular composition represents a potential source of confounding. The original study generating the analyzed dataset extensively characterized immune infiltration through deconvolution-based approaches and immunohistochemistry analyses, reporting comparable immune infiltration levels between the HCC and NT subsets considered here [23]. Our complementary evaluation of immune- and inflammation-related transcriptional signatures further supported broadly equivalent inflammatory profiles between the HCC and NT cohorts. Nevertheless, these observations do not exclude more subtle differences in cellular composition or cell-type-specific HERV expression, which cannot be resolved using bulk transcriptomic data alone. Third, although exploratory covariate analyses suggested limited effects of gender imbalance and viral etiology on the major HERV transcriptional signatures, the relatively small and uneven composition of the HCC cohort represents an additional important limitation. HBV-associated tumors constituted the largest etiologically defined subgroup, whereas only six HCV-associated cases and a limited number of non-viral tumors were available. Consequently, the etiology-related analyses should be regarded as exploratory and do not provide sufficient statistical power to establish HCV-specific or non-viral HERV signatures, nor to conclude that HBV- and HCV-associated HCC share equivalent HERV transcriptional profiles. Although covariate-adjusted analyses indicated that the major HCC-associated HERV signature was largely retained after adjustment for viral etiology, this finding primarily reflects the structure of the present cohort and should not be generalized to etiologically balanced HCC populations. Larger independent cohorts with adequate representation of HBV-, HCV-, and non-B/non-C HCC will be required to determine whether distinct etiological contexts are associated with specific HERV transcriptional patterns. Similarly, the limited number of samples within individual G1–G3 subgroups reduces the power to identify grade-associated HERV differences and requires caution when interpreting the increasing numbers of DE HERVs observed across grades. The aggregate-signature analyses provide additional support for a grade-associated relationship by evaluating the same fixed sets of HERV loci across individual samples; however, they do not overcome the limitations imposed by the cross-sectional design and relatively small grade-specific groups. These findings should therefore be considered grade-associated transcriptional patterns within the analyzed cohort rather than evidence of longitudinal changes during tumor progression. Likewise, the exploratory ROC analyses were performed in the same dataset used for HERV discovery and candidate selection and therefore cannot establish diagnostic or biomarker validity. Independent cohorts, ideally including appropriately matched non-tumoral and early-stage HCC samples, will be required to determine the reproducibility and clinical performance of these candidate loci. Finally, the present study is fundamentally correlative in nature. The observed associations between HERV loci, nearby cellular genes, and pathway enrichment profiles should therefore not be interpreted as evidence of direct regulatory or functional interactions. Rather, these analyses provide a transcriptomic framework for identifying HERV loci that may warrant future mechanistic investigation. Approaches based on single-cell transcriptomics, spatial transcriptomics, epigenomic profiling, and functional perturbation experiments will be necessary to define the cell-type specificity, regulatory mechanisms, and potential biological impact of HERV transcription in HCC.

Overall, the present study provides a comprehensive characterization of HERV transcriptional modulation in a cohort predominantly composed of viral-associated (particularly HBV-associated) HCC and identifies both broadly HCC-associated and histological-grade-associated HERV signatures embedded within the broader transcriptomic remodeling of liver tumorigenesis. The identification of candidate transcriptionally active HERV-derived loci and transcripts associated with inflammatory, metabolic, and proliferative programs highlights the complexity of locus-specific HERV activity in HCC tissues and provides a basis for future studies investigating their possible contribution to liver cancer biology and their synergy with viral hepatitis infections.

## CRediT authorship contribution statement

**Nicole Grandi:** Writing – review & editing, Writing – original draft, Supervision, Methodology, Investigation, Formal analysis, Data curation, Conceptualization. **Elena Soddu:** Writing – review & editing, Writing – original draft, Investigation, Formal analysis, Data curation. **Saili Chabukswar:** Writing – review & editing, Methodology, Formal analysis, Data curation. **Sante Scognamiglio:** Writing – review & editing, Methodology. **Yun Yen:** Writing – review & editing. **Ching-Hsuan Liu:** Writing – review & editing. **Liang-Tzung Lin:** Writing – review & editing, Supervision. **Enzo Tramontano:** Writing – review & editing, Supervision.

## Declaration of competing interest

The authors declare that they have no known competing financial interests or personal relationships that could have appeared to influence the work reported in this paper.

## Data Availability

Data will be made available on request.

## Appendix A Supplementary data

Multimedia component 1 Supplementary Figure S1: **Unsupervised clustering analyses based on HERV expression with indication of HCC etiologies.** Panel a) Sample-to-sample distance heatmap generated according to pairwise distance in HERV expression among samples. Samples' color indicates HCC etiologies: HBV = blue, HCV = violet, nonBnonC = green, NASH/MASLD = brown, Alcoholic = yellow, NA = orange. NT samples are indicated in red. Panel b) Principal Component Analysis (PCA) of HERV expression variance. PC1 (x-axis) and PC2 (y-axis) explain HERV transcriptional variance along with their percentage value. Samples are represented with colors accounting for HCC etiologies, while shapes account for tumor presence/absence. * = Alcoholic and NASH-induced outliers.

Multimedia component 2 Supplementary Figure S2: **Transcriptional signature of differentially expressed genes in tumoral, non-tumoral, and healthy liver samples.** Panel a) Heatmap of the TPM of DE genes among the dataset HCC and NT samples (GSE HCC and GSE NT, respectively) and the GTEx healthy liver controls. Column and row dendrograms show samples and genes' clustering, respectively. Genes are clustered according to their modulation in HCC (Cluster 1 = upregulated, Cluster 2 = downregulated). Panel b) Gene Enrichment dot plot of the 5 most enriched pathways among GO Biological Process 2025 library [31] according to p-adj ≤ 0.01 for Cluster 1 and Cluster 2 genes. Dot color represents -log10(p-adj) values, and dot size the ratio between the number of DE genes from the biological process and the total number of genes belonging to that process. Panel c) Dot plots showing transcript levels (TPM) of the top 20 DEL- (above) and top 20 DEL + genes (below) across GSE HCC, GSE NT, and GTEx HC.

Multimedia component 3 Supplementary Figure S3: **Overview of innate immune genes transcriptional levels.** Heatmap of the TPM of ∼1100 genes involved in innate immunity and inflammation (immuneDB) among the dataset HCC and NT samples (GSE HCC and GSE NT, respectively) and the GTEx healthy liver controls. Column and row dendrograms show samples and genes' clustering, respectively.

Multimedia component 4 Supplementary Figure S4: **Evaluation of the impact of biological covariates on HERV expression.** Panels A to D show the first 6 principal components (PC1 – PC6) accounting for HERV transcriptional variance across the samples. In each plot, samples are colored according to a different variable, including tumor condition (A), gender (B), viral etiology (C), and tumor grade (D). Each plot row reports the PCs on the y axis, along with their percentage value and the columns the PCs on x axis (PC2 to PC6, from left to right). Panel E) Triple Venn Diagram showing the DE HERVs identified according to the tumor condition (HCC) and including gender or viral etiology covariates to this main design.

Multimedia component 5 Supplementary Figure S5: **HERV modulation according to the different tumor grades** Heatmaps of expression of the 16 DE HERVs in common across all grades (a) and of the DE HERVs specifically modulated in the G2 (45, b), G3 (68, c), and HCC-ICC comorbidity (12, d) tumor samples.
