# Supplementary material for: Locus-specific endogenous retroviral transcriptional modulation in viral hepatitis–driven hepatocellular carcinoma
Source: Tumour Virus Res. 2026 Oct 29;22:200352. doi: 10.1016/j.tvr.2026.200352 (PMC13756109; doi:10.1016/j.tvr.2026.200352)
Supplement: Supplementary file 1 [file mmc1.pdf]

A

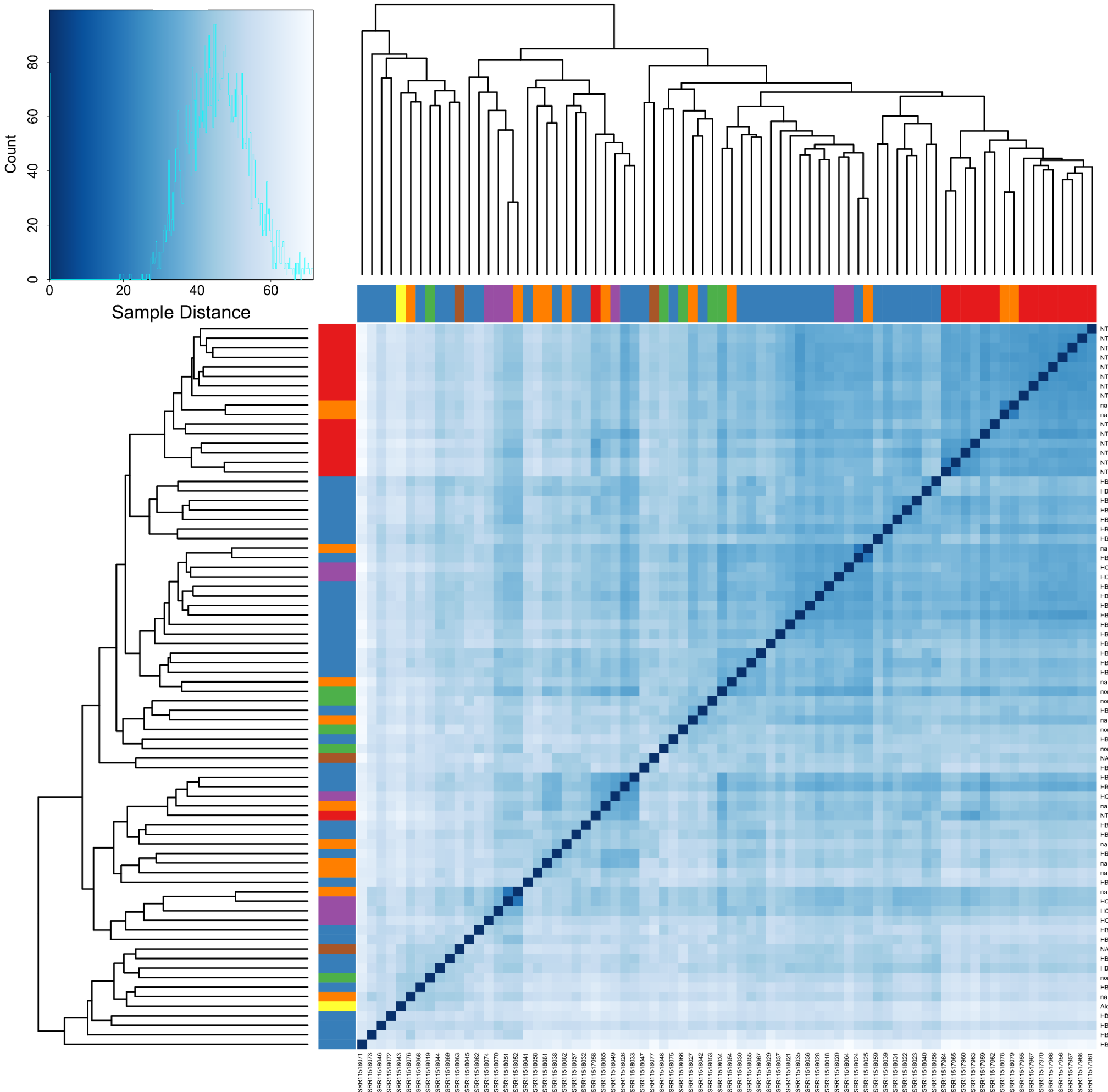
Count
Sample Distance
0
20
40
60
80
NT
na
HBV
HCV
nonBnonC
NASH
Alcoholic

B

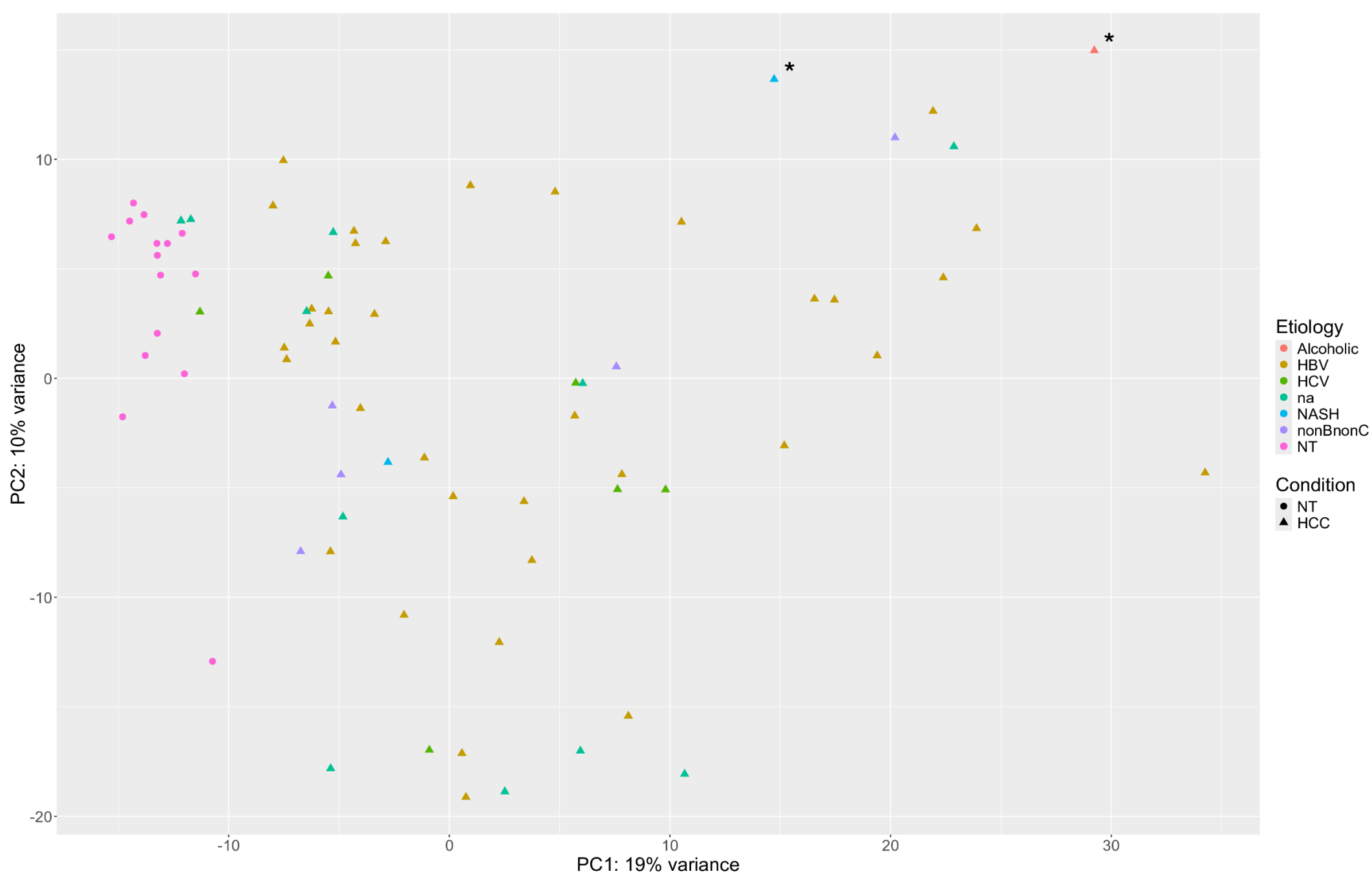
PC1: 19% variance
PC2: 10% variance
Etiology
Alcoholic
HBV
HCV
na
NASH
nonBnonC
NT
Condition
NT
HCC
*
*
