# Supplementary material for: Locus-specific endogenous retroviral transcriptional modulation in viral hepatitis–driven hepatocellular carcinoma
Source: Tumour Virus Res. 2026 Oct 29;22:200352. doi: 10.1016/j.tvr.2026.200352 (PMC13756109; doi:10.1016/j.tvr.2026.200352)
Supplement: Supplementary file 2 [file mmc2.pdf]

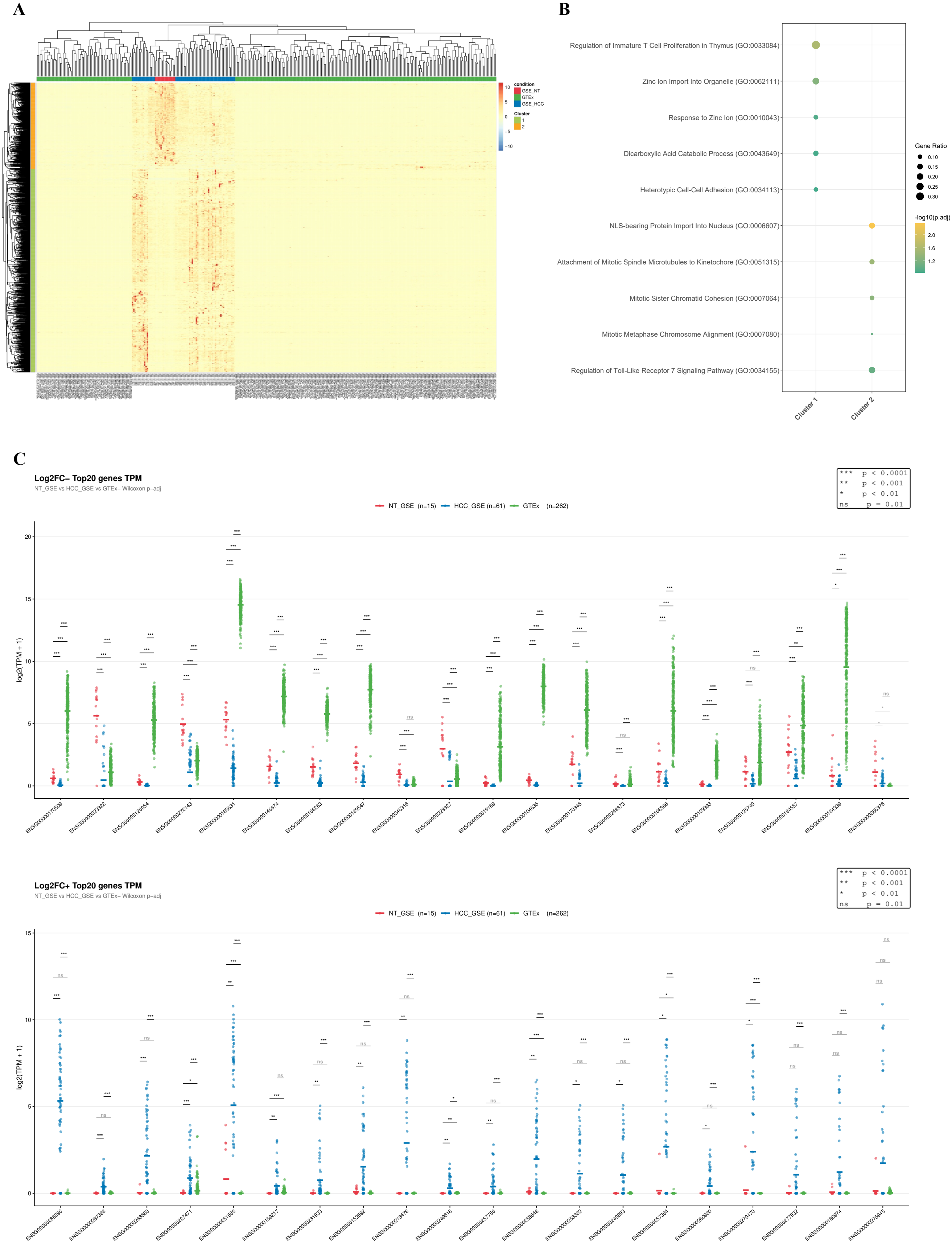
A
B
C
condition
GSE_NT
GTEx
GSE_HCC
Cluster
1
2
Regulation of Immature T Cell Proliferation in Thymus (GO:0033084)
Zinc Ion Import Into Organelle (GO:0062111)
Response to Zinc Ion (GO:0010043)
Dicarboxylic Acid Catabolic Process (GO:0043649)
Heterotypic Cell-Cell Adhesion (GO:0034113)
NLS-bearing Protein Import Into Nucleus (GO:0006607)
Attachment of Mitotic Spindle Microtubules to Kinetochore (GO:0051315)
Mitotic Sister Chromatid Cohesion (GO:0007064)
Mitotic Metaphase Chromosome Alignment (GO:0007080)
Regulation of Toll-Like Receptor 7 Signaling Pathway (GO:0034155)
Gene Ratio
-log10(p.adj)
Cluster 1
Cluster 2
Log2FC– Top20 genes TPM
NT_GSE vs HCC_GSE vs GTEx– Wilcoxon p-adj
NT_GSE (n=15)
HCC_GSE (n=61)
GTEx (n=262)
*** p < 0.0001
** p < 0.001
* p < 0.01
ns p = 0.01
log2(TPM + 1)
Log2FC+ Top20 genes TPM
NT_GSE vs HCC_GSE vs GTEx– Wilcoxon p-adj
