# Supplementary material for: Locus-specific endogenous retroviral transcriptional modulation in viral hepatitis–driven hepatocellular carcinoma
Source: Tumour Virus Res. 2026 Oct 29;22:200352. doi: 10.1016/j.tvr.2026.200352 (PMC13756109; doi:10.1016/j.tvr.2026.200352)
Supplement: Supplementary file 4 [file mmc4.pdf]

A

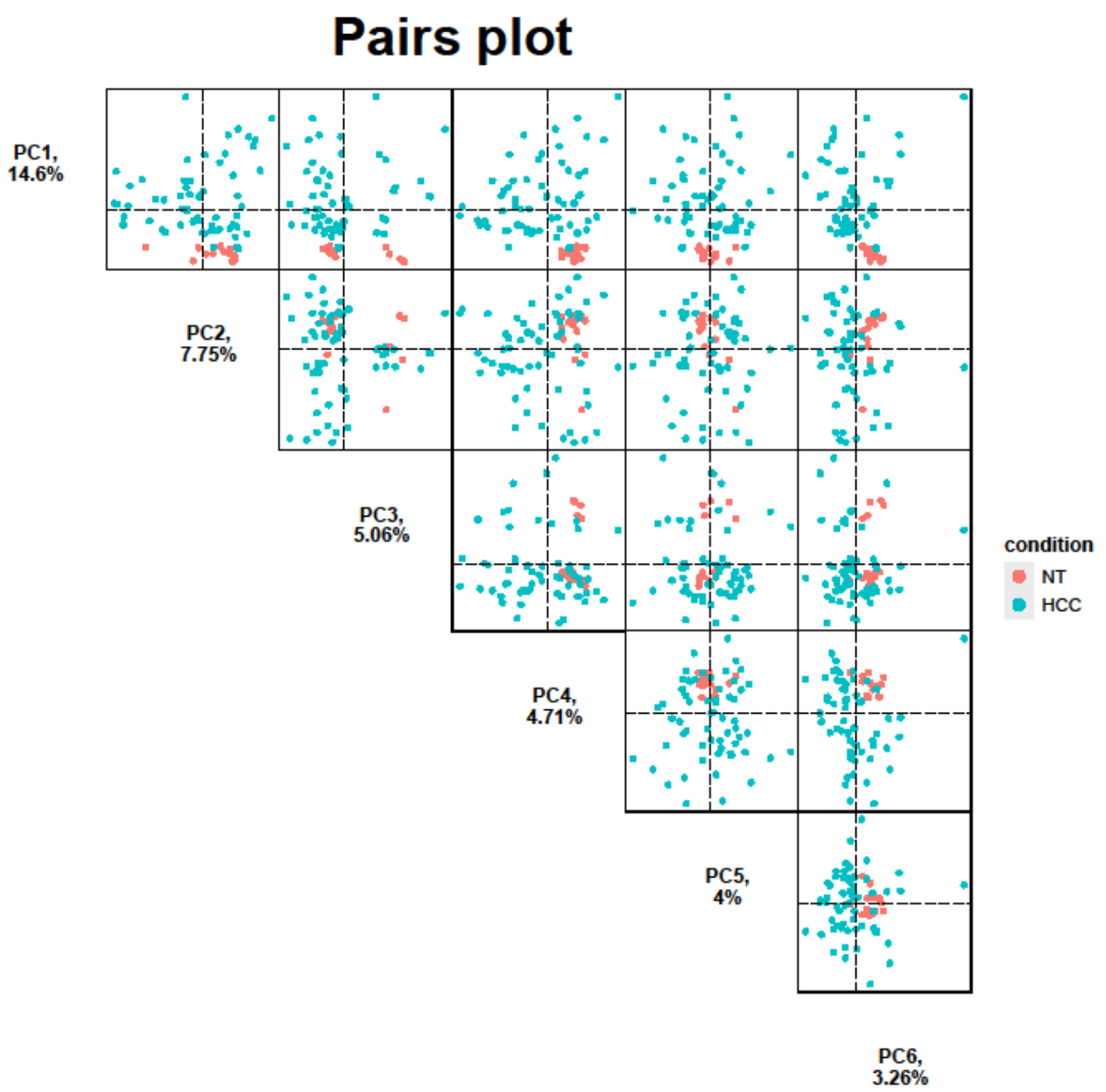
Pairs plot
PC1, 14.6%
PC2, 7.75%
PC3, 5.06%
PC4, 4.71%
PC5, 4%
PC6, 3.26%
condition
NT
HCC

B

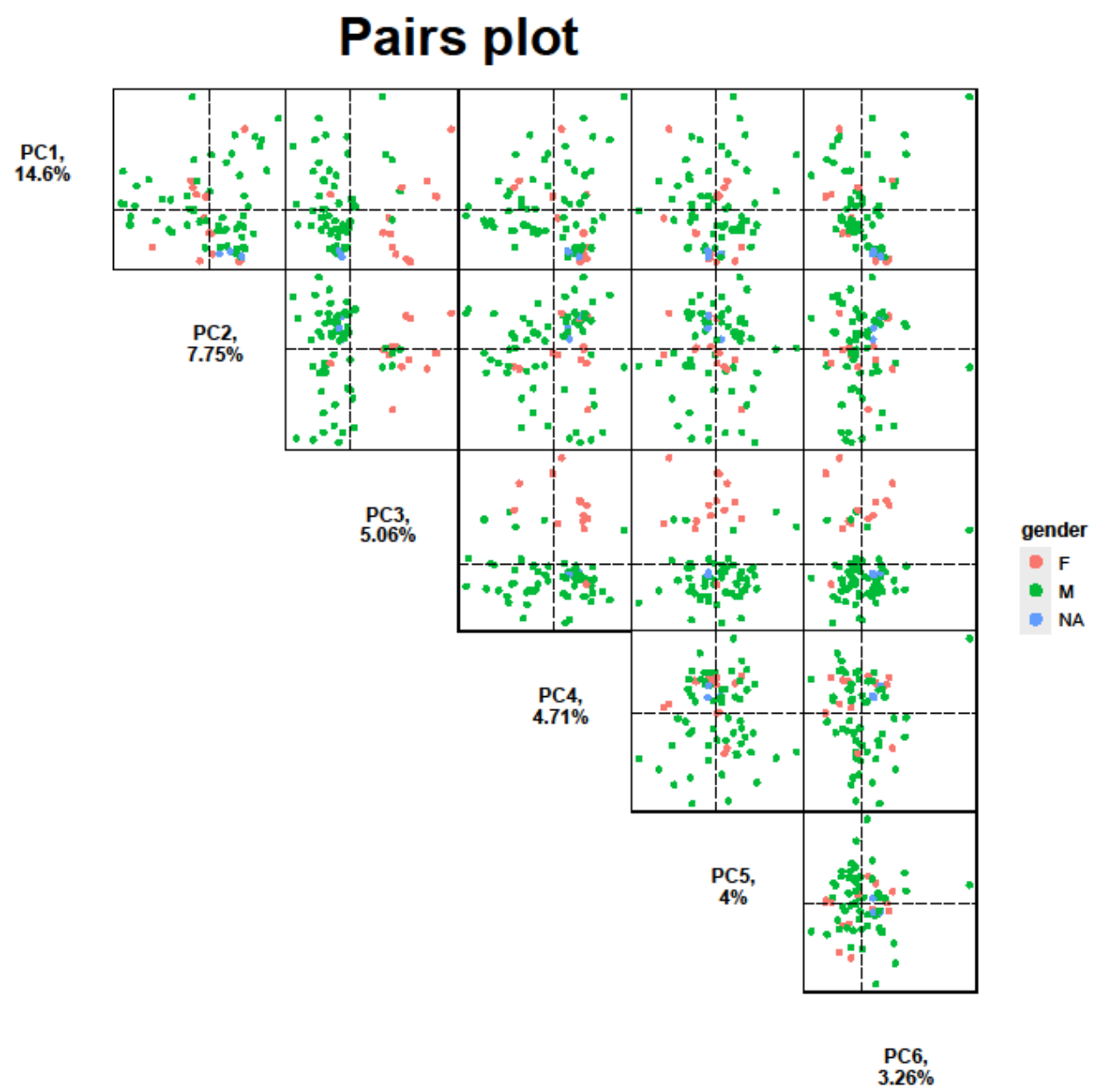
Pairs plot
PC1, 14.6%
PC2, 7.75%
PC3, 5.06%
PC4, 4.71%
PC5, 4%
PC6, 3.26%
gender
F
M
NA

C

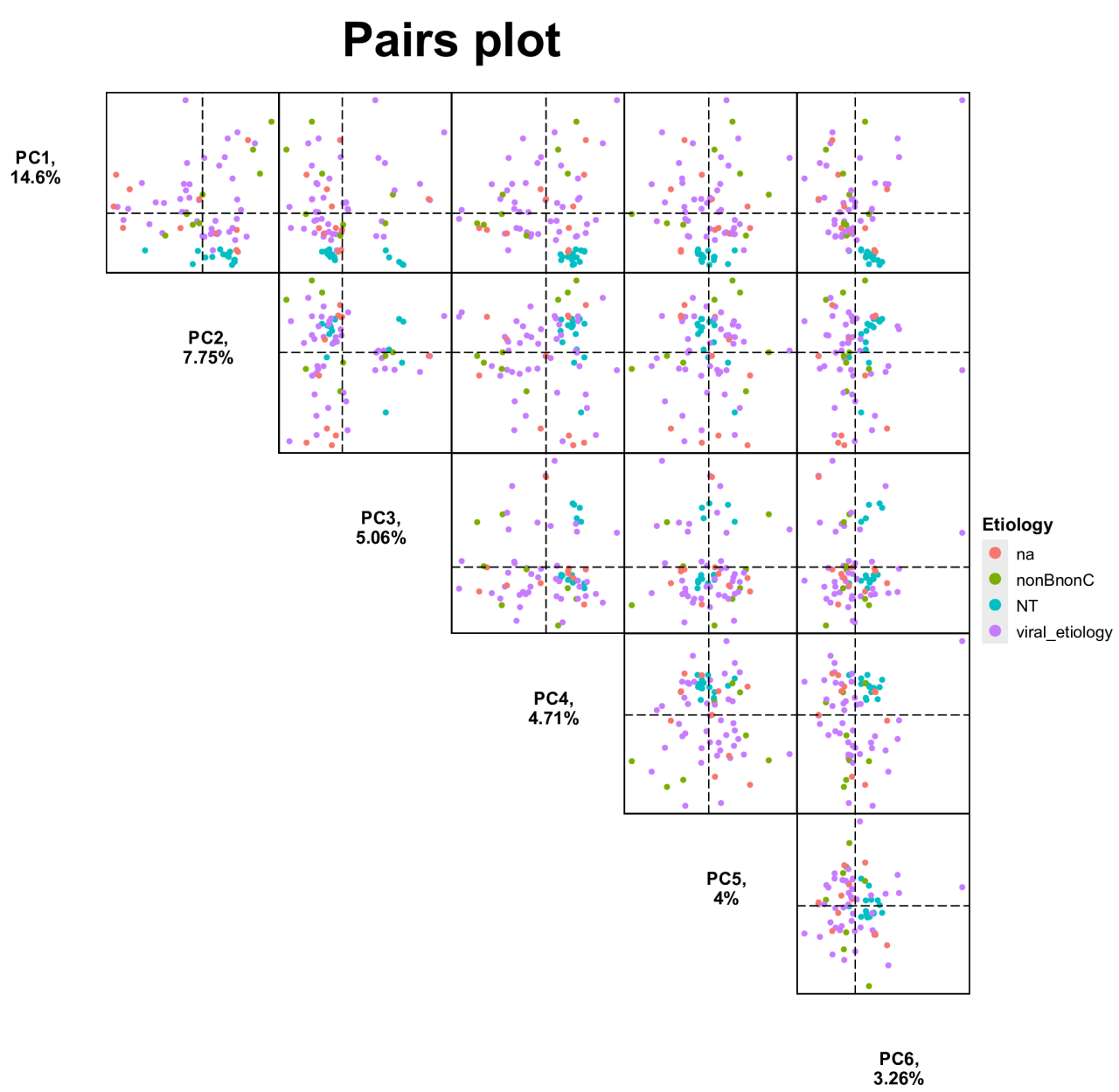
Pairs plot
PC1, 14.6%
PC2, 7.75%
PC3, 5.06%
PC4, 4.71%
PC5, 4%
PC6, 3.26%
Etiology
na
nonBnonC
NT
viral_etiology

D

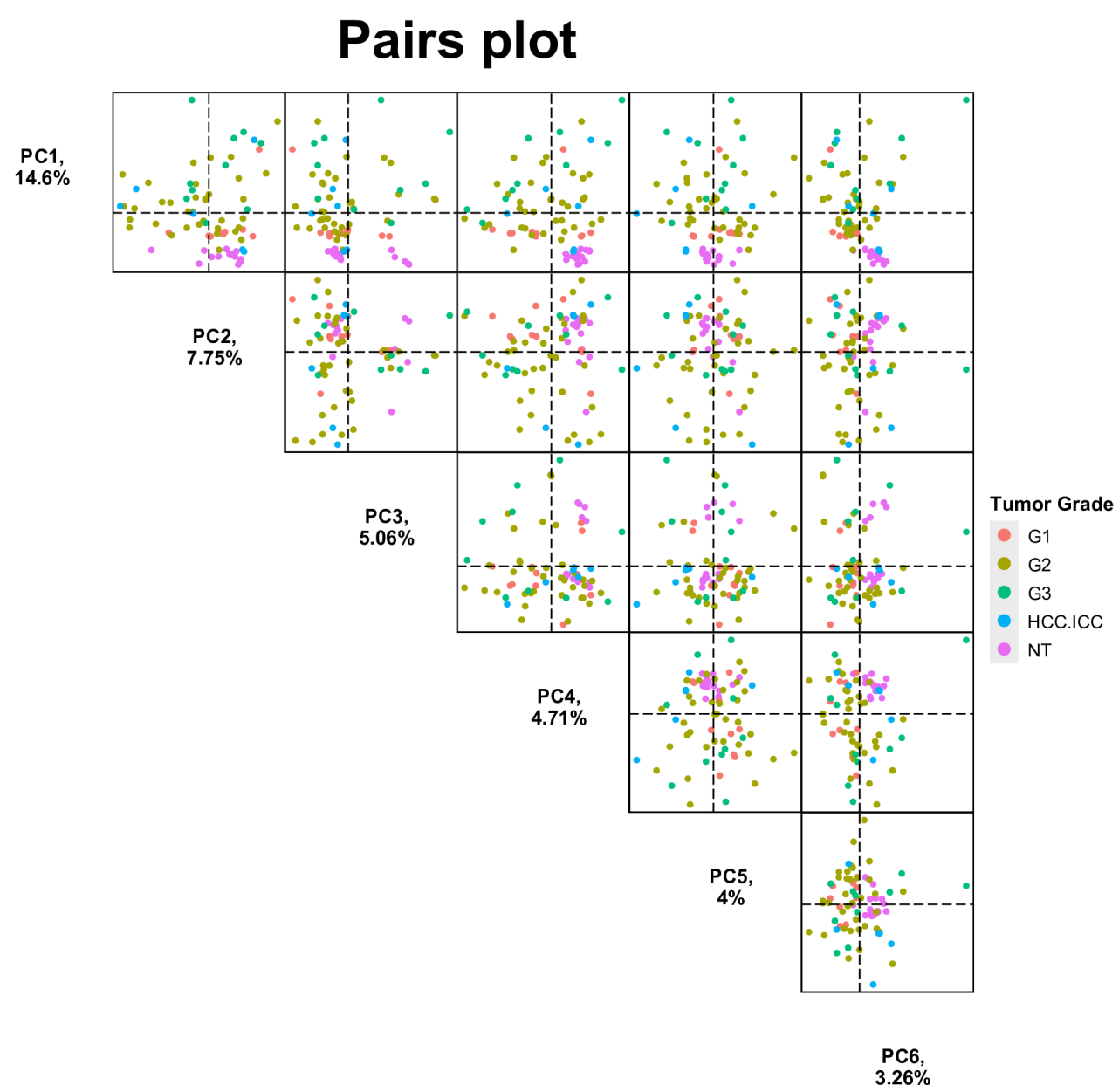
Pairs plot
PC1, 14.6%
PC2, 7.75%
PC3, 5.06%
PC4, 4.71%
PC5, 4%
PC6, 3.26%
Tumor Grade
G1
G2
G3
HCC.ICC
NT

E

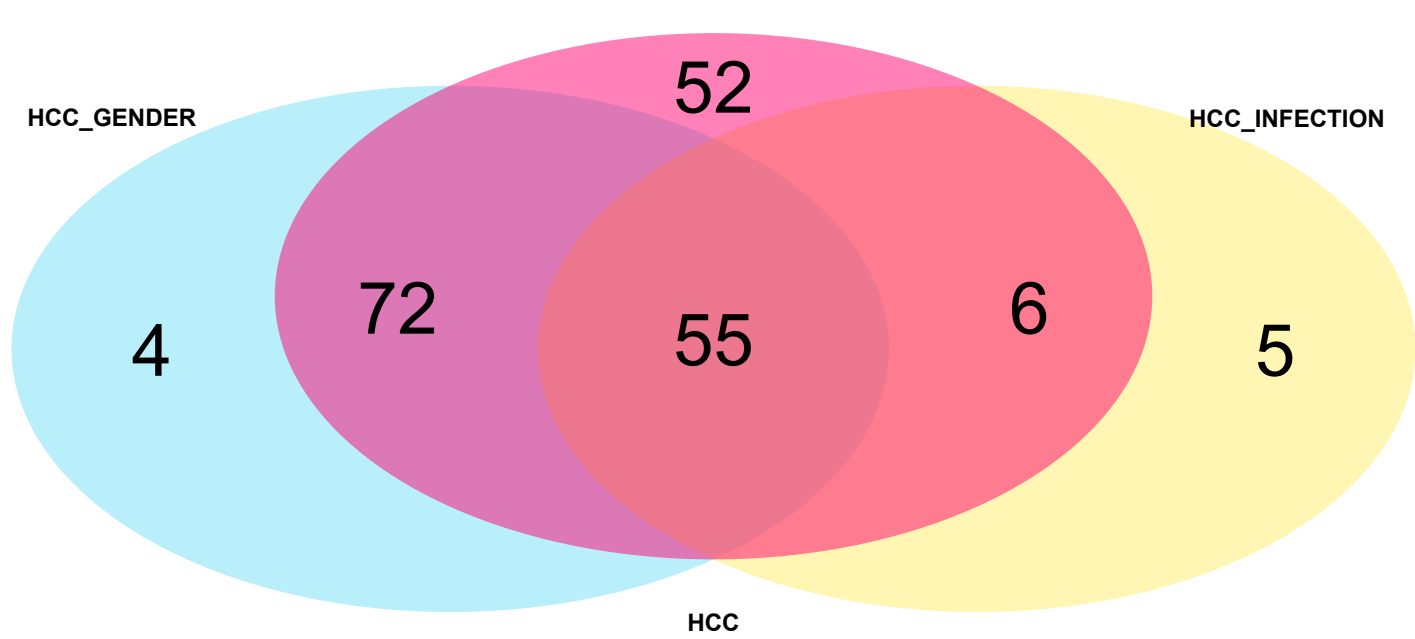
HCC_GENDER
HCC_INFECTION
HCC
52
4
72
55
6
5
