# Supplementary material for: Locus-specific endogenous retroviral transcriptional modulation in viral hepatitis–driven hepatocellular carcinoma
Source: Tumour Virus Res. 2026 Oct 29;22:200352. doi: 10.1016/j.tvr.2026.200352 (PMC13756109; doi:10.1016/j.tvr.2026.200352)
Supplement: Supplementary file 5 [file mmc5.pdf]

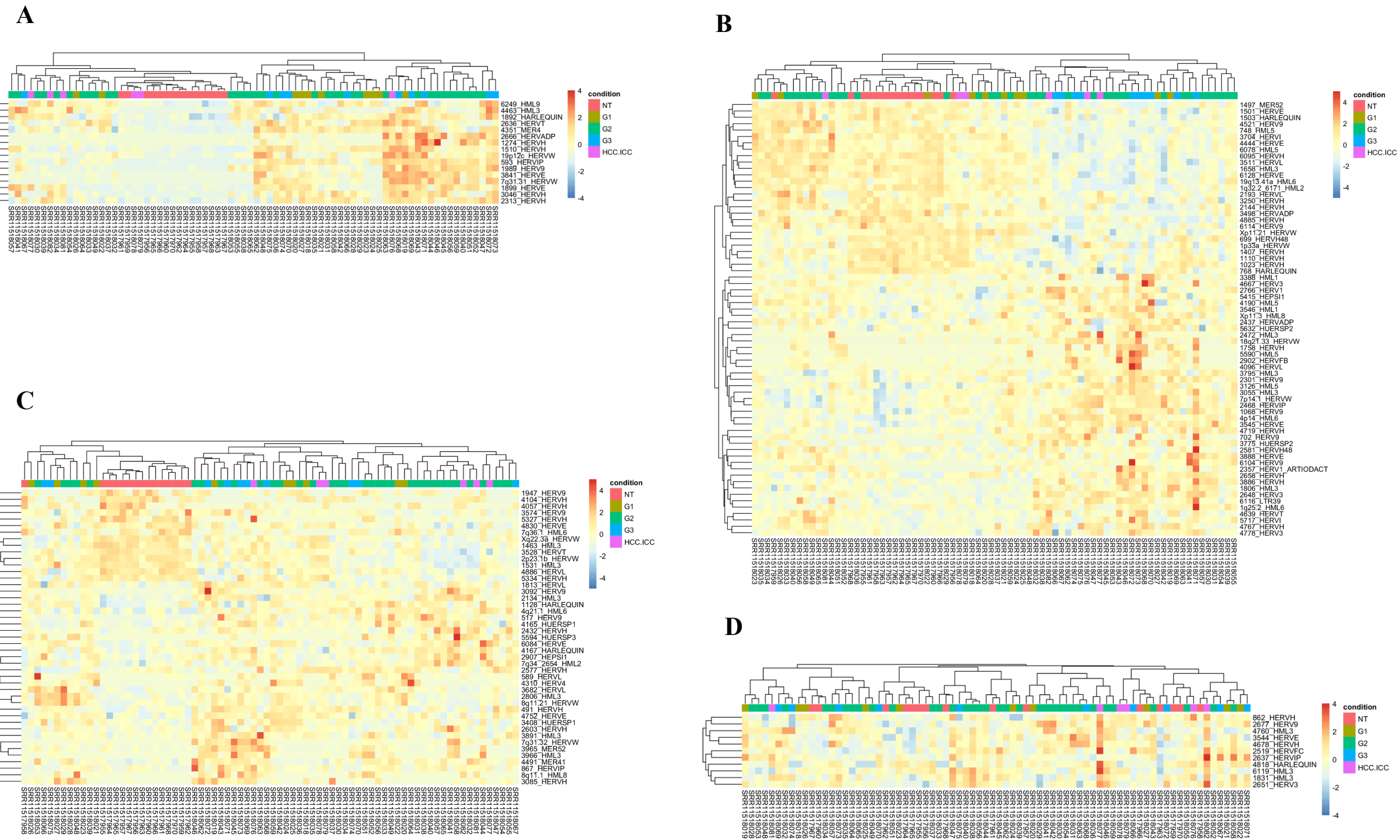
A
condition
NT
G1
G2
G3
HCC.ICC
4
2
0
-2
-4
6249_HML9
4463_HML3
1892_HARLEQUIN
2636_HERVT
4351_MER4
2666_HERVADP
1274_HERVH
1510_HERVH
19p12c_HERVW
593_HERVIP
1989_HERV9
3841_HERVE
7q31.31_HERVW
1899_HERVE
3046_HERVH
2313_HERVH
B
1497_MER52
1501_HERVE
1503_HARLEQUIN
4521_HERV9
748_HML5
3704_HERVI
4444_HERVE
6078_HML5
6095_HERVH
3511_HERVL
1658_HML3
6128_HERVE
19q13.41a_HML6
1q32.2_6171_HML2
2193_HERVL
3250_HERVH
2144_HERVH
3498_HERVADP
4885_HERVH
6114_HERV9
Xp11.21_HERVW
699_HERVH48
1p33a_HERVW
1407_HERVH
1110_HERVH
1023_HERVH
768_HARLEQUIN
3388_HML1
4667_HERV3
2766_HERV1
5415_HEPSI1
4190_HML5
3546_HML1
Xp11.3_HML8
2437_HERVADP
5632_HUERSP2
2472_HML3
18q21.33_HERVW
1758_HERVH
5590_HML5
2902_HERVFB
4096_HERVL
3795_HML3
2301_HERV9
3126_HML5
3055_HML3
7p14.1_HERVW
2468_HERVIP
1066_HERV9
4p14_HML6
3545_HERVE
4719_HERVH
702_HERV9
3775_HUERSP2
2581_HERVH48
3888_HERVE
6104_HERV9
2357_HERV1_ARTIODACT
2658_HERVH
3886_HERVH
1806_HML3
2648_HERV3
6116_LTR39
1q25.2_HML6
4639_HERVT
5717_HERVI
4767_HERVH
4778_HERV3
C
1947_HERV9
4104_HERVH
4057_HERVH
3574_HERV9
5327_HERVH
4830_HERVE
7q36.1_HML6
Xq22.3a_HERVW
1463_HML3
3528_HERVT
2p23.1b_HERVW
1531_HML3
4886_HERVL
5334_HERVH
1813_HERVL
3092_HERV9
2134_HML3
1128_HARLEQUIN
4q21.1_HML6
517_HERV9
4165_HUERSP1
2432_HERVH
5594_HUERSP3
6084_HERVE
4167_HARLEQUIN
2907_HEPSI1
7q34_2654_HML2
2577_HERVH
589_HERVL
4310_HERV4
3682_HERVL
2806_HML3
8q11.21_HERVW
491_HERVH
4752_HERVE
3408_HUERSP1
2603_HERVH
3891_HML3
7q31.32_HERVW
3965_MER52
3966_HML3
4491_MER41
867_HERVIP
8q11.1_HML8
3085_HERVH
D
862_HERVH
2677_HERV9
4760_HML3
3544_HERVE
4678_HERVH
2519_HERVFC
2637_HERVIP
4818_HARLEQUIN
6119_HML3
1831_HML3
2651_HERV3
