# Supplementary material for: Locus-specific endogenous retroviral transcriptional modulation in viral hepatitis–driven hepatocellular carcinoma
Source: Tumour Virus Res. 2026 Oct 29;22:200352. doi: 10.1016/j.tvr.2026.200352 (PMC13756109; doi:10.1016/j.tvr.2026.200352)
Supplement: Supplementary file 6 [file mmc6.docx]

| **SAMPLE ID** | **AGE** | **GENDER** | **HCC** | **G1-G6** | **ICC** | **ETIOLOGY** |
| --- | --- | --- | --- | --- | --- | --- |
| 11517955 | 17 | M | NP | NP | NP | NP |
| 11517956 | 54 | M | NP | NP | NP | NP |
| 11517957 | 64 | M | NP | NP | NP | NP |
| 11517958 | 50 | F | NP | NP | NP | NP |
| 11517959 | 73 | M | NP | NP | NP | NP |
| 11517960 | 73 | F | NP | NP | NP | NP |
| 11517961 | 76 | M | NP | NP | NP | NP |
| 11517962 | NA | NA | NP | NP | NP | NP |
| 11517963 | 76 | F | NP | NP | NP | NP |
| 11517964 | 31 | F | NP | NP | NP | NP |
| 11517965 | 31 | F | NP | NP | NP | NP |
| 11517966 | 54 | M | NP | NP | NP | NP |
| 11517967 | 66 | M | NP | NP | NP | NP |
| 11517968 | NA | NA | NP | NP | NP | NP |
| 11517969 | NA | NA | NP | NP | NP | NP |
| 11517970 | NA | NA | NP | NP | NP | NP |
| 11518018 | 66 | M | HCC | G1 | NP | HBV |
| 11518019 | 64 | M | HCC | G1 | NP | nonBnonC |
| 11518020 | 60 | M | HCC | G1 | NP | HCV |
| 11518021 | 53 | M | HCC | G1 | NP | HBV |
| 11518022 | 43 | F | HCC | G1 | NP | HBV |
| 11518023 | 52 | F | HCC | G1 | NP | HBV |
| 11518024 | 58 | M | HCC | G1 | NP | HBV |
| 11518025 | 58 | M | HCC | G1 | NP | NA |
| 11518026 | 60 | M | HCC | G1 | NP | HBV |
| 11518027 | 64 | M | HCC | G1 | NP | NA |
| 11518028 | 41 | M | HCC | G2 | NP | HBV |
| 11518029 | 53 | M | HCC | G2 | NP | HBV |
| 11518030 | 39 | M | HCC | G2 | NP | HBV |
| 11518031 | 50 | F | HCC | G2 | NP | HBV |
| 11518032 | 48 | M | HCC | G2 | NP | HBV |
| 11518033 | 49 | M | HCC | G2 | NP | HBV |
| 11518034 | 52 | M | HCC | G2 | NP | nonBnonC |
| 11518035 | 58 | M | HCC | G2 | NP | HBV |
| 11518036 | 58 | M | HCC | G2 | NP | HBV |
| 11518037 | 42 | M | HCC | G2 | NP | HBV |
| 11518038 | 58 | M | HCC | G2 | NP | HBV |
| 11518039 | 36 | M | HCC | G2 | NP | HBV |
| 11518040 | 40 | M | HCC | G2 | NP | HBV |
| 11518041 | 56 | M | HCC | G2 | NP | HBV |
| 11518042 | 56 | M | HCC | G2 | NP | HBV |
| 11518043 | 64 | M | HCC | G2 | NP | Alcoholic |
| 11518044 | 57 | M | HCC | G2 | NP | HBV |
| 11518045 | 47 | M | HCC | G2 | NP | HBV |
| 11518046 | 78 | M | HCC | G2 | NP | HBV |
| 11518047 | 51 | M | HCC | G2 | NP | HBV |
| 11518048 | 57 | M | HCC | G2 | NP | nonBnonC |
| 11518049 | 61 | M | HCC | G2 | NP | HCV |
| 11518050 | 70 | M | HCC | G2 | NP | nonBnonC |
| 11518051 | 62 | F | HCC | G2 | NP | HCV |
| 11518052 | 62 | F | HCC | G2 | NP | NA |
| 11518053 | 53 | M | HCC | G2 | NP | nonBnonC |
| 11518054 | 49 | M | HCC | G2 | NP | NA |
| 11518055 | 57 | M | HCC | G2 | NP | HBV |
| 11518056 | 75 | F | HCC | G2 | NP | HBV |
| 11518057 | 60 | M | HCC | G2 | NP | HBV |
| 11518058 | 57 | M | HCC | G2 | NP | NA |
| 11518059 | 46 | M | HCC | G2 | NP | HBV |
| 11518060 | 52 | F | HCC | G2 | NP | HBV |
| 11518061 | 50 | M | HCC | G2 | NP | HBV |
| 11518062 | 54 | M | HCC | G2 | NP | HBV |
| 11518063 | 49 | M | HCC | G2 | NP | NASH |
| 11518064 | 66 | M | HCC | G2 | NP | HCV |
| 11518065 | 44 | M | HCC | G2 | NP | NA |
| 11518066 | 70 | M | HCC | G3 | NP | nonBnonC |
| 11518067 | 48 | M | HCC | G3 | NP | HBV |
| 11518068 | 62 | M | HCC | G3 | NP | HBV |
| 11518069 | 38 | M | HCC | G3 | NP | HBV |
| 11518070 | 75 | F | HCC | G3 | NP | HCV |
| 11518071 | 65 | M | HCC | G3 | NP | HBV |
| 11518072 | 51 | M | HCC | G3 | NP | HBV |
| 11518073 | 49 | F | HCC | G3 | NP | HBV |
| 11518074 | 70 | F | HCC | G3 | NP | HCV |
| 11518075 | 65 | M | HCC | G3 | NP | HBV |
| 11518076 | 57 | M | HCC | NA | w/ ICC | NA |
| 11518077 | 55 | M | HCC | NA | w/ ICC | NASH |
| 11518078 | 48 | M | HCC | NA | w/ ICC | NA |
| 11518079 | 48 | M | HCC | NA | w/ ICC | NA |
| 11518080 | 61 | M | HCC | NA | w/ ICC | NA |
| 11518081 | 44 | M | HCC | NA | w/ ICC | NA |
| 11518082 | 36 | M | HCC | NA | w/ ICC | NA |

**Table S1: Overview of GSE148355 dataset.** The characteristics of the individual samples are reported, including sample ID, age, gender, HCC presence, G1-G3 tumor stage, comorbidity with ICC and tumor etiology. NP = not present; NA = not available.
