# Supplementary material for: Locus-specific endogenous retroviral transcriptional modulation in viral hepatitis–driven hepatocellular carcinoma
Source: Tumour Virus Res. 2026 Oct 29;22:200352. doi: 10.1016/j.tvr.2026.200352 (PMC13756109; doi:10.1016/j.tvr.2026.200352)
Supplement: Supplementary file 7 [file mmc7.docx]

| **Study** | **Cohort** | **RNAseq** | **Methodology** | **HERV Modulation in HCC** | **Prognostic & Molecular Classification** | **In vitro findings** |
| --- | --- | --- | --- | --- | --- | --- |
| **This study** | **Korean HCC cohort (GSE148355)**  • 61 T tissues (including 36 HBV  6 HCV cases)  • 15 NT tissues | **Total RNA**  2×100-bp PE, 100M  (Illumina HiSeq 2000) | • **STAR** alignment  • **htseqCount** with **in house HERVdb** (hg38)  • **DESeq2** for DE analysis (\|log2FC\|>1, padj<0.01) | • **185 DE HERVs**  (145 up, 40 down)  • **16 DE HERVs** upregulated in all histological grades | • Identified 16 HERV loci consistently upregulated across all HCC histological grades, with aggregate expression significantly associated with grade.  • Prioritized candidate loci showing the highest exploratory discriminatory performance | - |
| **Chang et al., *Cancers* (2023)** | **254 Taiwanese HCC patients**  • 254 T and 34 matched adjacent NT tissues  • 102 HBV and 7 HCV positive | **Total RNA**  2×151-bp PE, 100–200M  (Illumina NovaSeq 6000) | • **DRAGEN** alignment  • **Telescope** (hg38)  • **DESeq2** for DE analysis (\|log2FC\|>1.5, padj<0.05) | • **3,357 DE HERVs** (3,161 up, 196 down)  • **180 DE HERVs** correlated with overall survival (OS) | • Classified HCC into **4 molecular subgroups**: *HERV-H1, -H2, -H3* (high activation, worse OS) and *HERV-L* (low activation, better OS)  • H subgroups correlated with advanced age, high AFP, *TP53* / *TERT* promoter / *LRP1B* mutations, copy-number losses (*KDM6A*, *TP53*, *PER1*) | • **Modulating alternative splicing** via BS008 adjusted survival-related HERV and host gene expressions in Huh-7 cells |
| **Chang et al., *BMC Microbiology* (2025)** | **Same 254 Taiwanese HCC patients** as in Chang et al., 2023 | **Total RNA**  2×151-bp PE, 100–200M  (Illumina NovaSeq 6000) | • **DRAGEN** alignment  • **HERV-Fcount** pipeline: combines **featureCounts** with **Telescope** GTF annotations (hg38)  • **DESeq2** for DE analysis (\|log2FC\|>1.5, padj<0.05) | • **3,731 DE HERVs** (3,536 up, 195 down)  • **180 DE HERVs** correlated with overall survival (OS) | • Classified HCC into the above **4 molecular subgroups**: worse survival (HERV-H1, -H3) associated with females, old age, high total bilirubin, high AFP, advanced ES grade, and chronic HBV infection  • Found **14 survival-related DE HERVs** whose nearby genes co-expressed and had survival significance in the same direction (11 positive, 3 negative) | • **TP53 knockdown** in HepG2 cells led to the dysregulation of 98 of the 206 survival-related HERVs and 71 nearby genes |
| **Igarashi et al., *JHEP Reports* (2025)** | **TCGA-LIHC**  • 242 primary HCC cases | **Poly(A)**  2×50-bp PE,  40–240M  (Illumina HiSeq 2000/2500) | • **STAR** for alignment  • **featureCounts** with **gEVE annotations** (hg38)  • **DESeq2** for DE analysis (\|log2FC\|>1)  • **ChIP-qPCR** to measure H3K9me3 and SETDB1-binding levels | • Strong **inverse correlation** between **SETDB1** expression and HERV expression  • **10 HERV elements** suppressed by SETDB1 **H3K9me3 deposition**  • **4 selected HERV elements** (chr 5, 12, 7, and X) for detailed analysis | • Low expression of the 4 elements associated with poor OS and disease-free survival (DFS)  • Combined expression (**"HERV score"**) as independent prognostic factor | • **SETDB1 knockdown** in human (HuH7, HLE) cell lines derepressed these 4 HERVs, triggering an **antiviral "viral mimicry" IFN-***α* **response** and upregulating tumor suppressors  • **SETDB1 knockdown** in mouse (Hepa1-6) cell lines reduced in vivo tumor growth with increased *CD*8+ T-cell infiltration |
| **Wen et al., *Mobile DNA* (2025)** | **TCGA-LIHC**  • 371 T and 50 matched adjacent NT tissues  • 33 HBV and 1 HCV positive | **Poly(A)**  2×50-bp PE, 40–240M  (Illumina HiSeq 2000/2500) | • **Bowtie2** for alignment  • **Telescope** (hg38)  • **DESeq2** for DE analysis (padj<0.1 or <0.05)  • **PathoScope 2.0** to infer HBV and HCV viral status  • **Spearman correlation** between *HERVH* and *TP53*/*TERT* expression  • **GSEA** to assess HERV family enrichment | • **216 DE HERVs**  (50 up, 166 down)  • **HERVE and HERV9** enriched among up, **HERVH** enriched among down HERVs | • top 100 DE HERVs separated patients into **HERV-Low** (254 tumor-only) and **HERV-High** (117 tumors, all 50 NT)  • "HERV-High" tumors (less severe downregulation) had **poorer OS**  • Selected HERV loci selectively upregulated by **HBV** (*HERVL40_5q12.3*) or **HCV** (*ERVLB4_7q21.11c*) | - |
